# A global profiling of uncapped mRNAs under cold stress reveals specific decay patterns and endonucleolytic cleavages in *Brachypodium distachyon*

**DOI:** 10.1186/gb-2013-14-8-r92

**Published:** 2013-08-30

**Authors:** Jingyu Zhang, Zhiwei Mao, Kang Chong

**Affiliations:** 1 Key Laboratory of Plant Molecular Physiology, Institute of Botany, Chinese Academy of Sciences, Beijing 100093, China

**Keywords:** mRNA stability, decapping, cold stress, PARE, *Brachypodium distachyon*

## Abstract

**Background:**

mRNA degradation is a critical factor in determining mRNA abundance and enables rapid adjustment of gene expression in response to environmental stress. The involvement of processing bodies in stress response suggests a role for decapping-mediated mRNA degradation. However, little is known about the role of mRNA degradation under stressful environmental conditions.

**Results:**

Here, we perform a global study of uncapped mRNAs, via parallel analysis of RNA ends (PARE), under cold stress in *Brachypodium distachyon*. Enrichment analysis indicates that degradation products detected by PARE are mainly generated by the decapping pathway. Endonucleolytic cleavages are detected, uncovering another way of modulating gene expression. PARE and RNA-Seq analyses identify four types of mRNA decay patterns. Type II genes, for which light-harvesting processes are over-represented in gene ontology analyses, show unchanged transcript abundance and altered uncapped transcript abundance. Uncapping-mediated transcript stability of light harvesting-related genes changes significantly in response to cold stress, which may allow rapid adjustments in photosynthetic activity in response to cold stress. Transcript abundance and uncapped transcript abundance for type III genes changes in opposite directions in response to cold stress, indicating that uncapping-mediated mRNA degradation plays a role in regulating gene expression.

**Conclusion:**

To our knowledge, this is the first global analysis of mRNA degradation under environmental stress conditions in *Brachypodium distachyon*. We uncover specific degradation and endonucleolytic cleavage patterns under cold stress, which will deepen our understanding of mRNA degradation under stressful environmental conditions, as well as the cold stress response mechanism in monocots.

## Background

Degradation of messenger RNAs (mRNAs) is one of the key factors determining mRNA abundance in a cell. Proper degradation of mRNA is crucial for the maintenance of cellular homeostasis and enables rapid and precise adjustment of gene expression in response to various environmental stimulus[1,2]. The study of mRNA degradation in plants has fallen behind compared with what has been done in other organisms and recent advances in this area provide us with only preliminary knowledge about plant RNA decay pathways[3-5]. For normal mRNAs, degradation is believed to initiate with deadenylation, the deadenylated mRNA can then either be degraded in the 3'-5' direction in a process mediated by the exosome or be decapped and then degraded in the 5'-3' direction[3]. Alternatively, mRNA decay in plants can be initiated with internal cleavage(s), for example, by miRNA- or siRNA-directed RISC degradation[6-9]. The mechanism of aberrant mRNAs degradation in plants is largely unknown, except for the identification of some key enzymes in the plant nonsense-mediated decay (NMD) pathway[10,11]. Obviously, our understanding of the degradation pathways in plants is still far from complete.

In eukaryotic cells, decapping is a critical step in mRNA turnover as it requires the stop of translation and the change of mRNAs into a non-translating state, which makes them accessible for further degradation. Decapping, to a large extent, competes with translation for the access to the 5' cap structure, which determines whether the mRNAs will be degraded or translated[12-15]. Recently, ribosomes are reported to be allowed to bind to mRNAs and complete translation before the transcript is ultimately degraded[16]. Messenger ribonucleoproteins (mRNPs) that are translationally repressed and associated with the decapping machinery can assemble into granules, named as processing bodies (PBs) [17,18]. The assembly of PBs occurs primarily in response to stress stimuli, and the size of PBs has been found to increase significantly under stress conditions[19], implying that PBs play an important role during plant stress responses. Furthermore, PBs co-localize with stress granules (SGs), suggesting that they probably interact with each other and function together in stress responses[19-21]. The significant increase of PBs in response to stress and their close connection with SGs indicate that mRNA decay, especially the decapping pathway, plays an important role in cellular stress responses[22,23]. However, little information is available for a complete view of mRNA degradation under stress conditions.

In this study, uncapped transcriptome has been analyzed with a deep sequencing approach, parallel analysis of RNA ends (PARE)[24]. Taking advantage of the modified 5' rapid amplification of cDNA ends (RACE) and high-throughput deep sequencing, PARE was developed to identify 5'-phosphorylated, polyadenylated RNAs[24]. In most cases, it was used to identify potential targets for miRNA-directed cleavages[25-28]. In mouse, this approach was employed to perform a global analysis of the RNA degradome[29,30]. This kind of application, as far as we know, was seldom reported in plant, except for a microarray study with similar experimental principle[31]. Here, this approach was employed to study mRNA degradation under cold stress condition in *Brachypodium distachyon*, a new monocot model plant [32]. Our studies will not only improve our understanding of mRNA degradation mechanism under stress conditions, but also provide deeper insight into how monocot plants respond to low temperature stress.

## Results

### Global analysis of the uncapped mRNAs under cold stress

To obtain a complete view of the uncapped transcriptome and understand its role in plant cold stress responses, PARE was performed to identify 5'-phosphorylated RNAs using high-throughput sequencing[24]. The *Brachypodium *diploid ecotype ABR5, with obviously higher cold tolerance and stronger vernalization requirement than the sequenced ecotype BD21 (Figure S1 in Additional file 1), was chosen for our analysis. Four PARE libraries were prepared. DC and DW were for *Brachypodium *seedlings with and without cold treatment, respectively. DC replicate and DW replicate were biological replicates for DC and DW (Table S1 in Additional file 2). For direct comparison, samples used for PARE analysis were prepared in the same manner and run side by side on Illumina GAIIx platform for high-throughput sequencing.

For all the four PARE libraries, over 10 million reads were generated through sequencing, respectively (Table S1 in Additional file 2). After removing sequences corresponding to known non-coding RNAs (rRNAs, tRNAs, small nuclear RNAs, and small nucleolar RNAs) and repeats/transposons, the remaining unique signatures were mapped to *Brachypodium *transcriptome[33]. For DW and DC libraries, 65.48% and 67.68% of the generated sequences could be mapped to annotated transcripts. For DW replicate and DC replicate libraries, 56.56% and 57.89% of the generated sequences could be mapped to annotated transcripts (Table S1 in Additional file 2). For direct comparison, the DC library was normalized to the DW library according to the population size of mapped transcripts (Table S1 in Additional file 2). Biological replicates libraries for DW and DC exhibited good reproducibility (Figure S2 in Additional file 1). DW and DC libraries were used for further analysis, and the obtained results were verified in their biological replicates.

About 12 thousand genes were found to be represented by PARE reads in the uncapped transcriptome. The most abundant uncapped transcript in the DW library was from a gene encoding chlorophyll a-b binding protein, whereas in the DC library, except for a protein with unknown function, it was from agene encoding cold acclimation protein (Table S2 in Additional file 2). When genes with the top 20 highest PARE reads were considered, the DW library was composed mainly of degradation products for chlorophyll-related genes (Table S2 in Additional file 2). After cold treatment, mRNA degradation products for cold-stress-related genes appeared in the DC library and accounted for a proportion of sequences with high sequencing reads, while the uncapped transcript abundance for chlorophyll-related genes was still high (Table S2 in Additional file 2). Cold stress was observed to exert a clear effect on the uncapped transcriptome. Overall, approximately half of uncapped transcripts showed obviously altered abundance (>5-fold) in the cold-stressed *Brachypodium *seedlings. Interestingly, several phytohormone-related genes showed significant up-regulation after cold treatment (Table S3 in Additional file 2).

It is interesting to note that a considerable portion of the unique reads for both DW and DC libraries were mapped to non-coding RNAs (ncRNAs), which has also been observed for PARE libraries generated from *Arabidopsis*, mouse and human[29,34,35]. No further analysis was performed on ncRNAs in this paper, which mainly focused on the degradation of mRNAs.

According to the principle of PARE analysis, only polyadenylated RNAs will be detected. Deadenylation precedes decapping in mRNA degradation pathways [3-5], which means some mRNAs, whose poly(A) tails length are too short (<18nt), can not be represented in the PARE library. Although the information about poly(A) tail length in plants is hardly available, mRNAs are generally believed to possess long poly(A) tails (about 250 nucleotides), which is shortened to dozens of nucleotides in deadenylation[36,37]. Only 18 adenosine residues of poly(A) tail are needed for PARE analysis [24], suggesting that the negative effect caused by deadenylation may be limited to a small range.

### Relating uncapping-mediated mRNA decay profiles to transcriptome

To globally analyze the contribution of mRNA decay to gene expression regulation, mRNA abundance was analyzed by RNA-Seq. Two cDNA libraries, with (RC) and without (RW) cold treatment, were prepared from *Brachypodium *seedlings. High-throughput sequencing was performed and the obtained results were verified by real-time RT-PCR (Figure S3 in Additional file 1). Cold stress had obvious effect on *Brachypodium *transcriptome. According to the results of RNA-Seq analysis, approximately one-third of genes showed significant changes (>5-fold) in their expression levels after cold treatment.

About eight thousand genes could be found in both PARE and RNA-Seq libraries. The PARE dataset was analyzed for degradation patterns, in conjunction with them RNA abundance data provided by RNA-Seq, with log_2 _fold change of ±1.5 as the threshold. Four major change patterns were identified (Table 1). For type I genes, the abundance of their transcripts and uncapped transcripts changed in the same direction after cold treatment. For genes in type II, uncapped transcripts showed clear changes after cold treatment, but their transcript abundance remained unchanged. For genes belonging to type III, the abundance of their transcripts and uncapped transcripts changed in the opposite direction after cold treatment. Transcripts of class IV genes showed clear changes in abundance after cold treatment, but their uncapped transcript abundance remained unchanged. About three-eighths of the genes belonged to type II group, while only one-eighth of genes were grouped as type III. The rest of the genes distributed almost equally between I and type IV (Table 1). Similar results were obtained for biological replicate libraries of DW and DC (data not shown).

**Table 1:** Four types of genes with different decay patterns in cold stress response.

Type	Gene number	Change after cold-treatment
I	2,136	D↑ R ↑; D↓ R↓
II	3,344	D↑ R **- **; D↓ R**- **
III	1,166	D↑ R ↓; D↓ R↑
IV	1,632	D**- **R ↑; D**- **R↓

I, II, III, and IV: different mRNA decay patterns classified according to the variation tend of uncapped transcript/transcript abundance in cold stress. D: uncapped transcript abundance indicated by PARE reads; R: transcript abundance indicated by RNA-Seq reads. Log_2 _ratio of±1.5 was selected as the threshold of change.

### Relationship between uncapping-mediated mRNA decay and gene function

**Figure 1:**
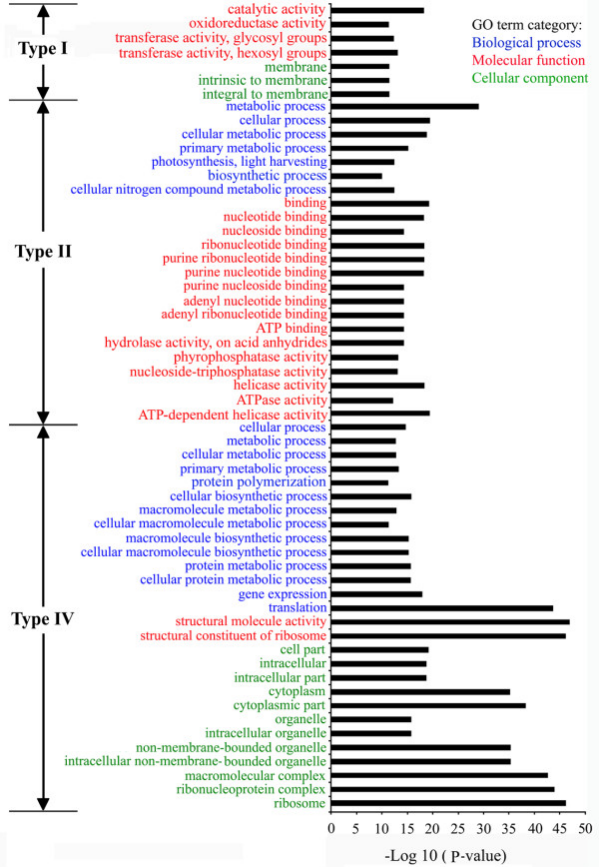
**Relationship between mRNA decay pattern and gene function**. Gene Ontology (GO) analysis was performed for the type I-IV genes using agriGO [109] which organizes information for molecular function, biological process, and cellular component categories. Significant GO terms (*P *value <1.00 E-10) were indicated for type I, type II, and type IV genes. No GO terms with *P *value <1.00 E-10 were identified for type III genes (more information in Figure S4 in Additional data file 3).

Gene Ontology (GO) analysis was performed for type I-IV genes using the GO database, which organizes information based on molecular function, biological process, and cellular component categories [38]. In the biological process category, obvious over-representation against background was detected for the light-harvesting process of photosynthesis and nitrogen compound metabolic process for the type II population (Figure 1). When just specific biological processes were considered, only light-harvesting-related genes involved in photosynthesis were significantly enriched in type II. Obvious enrichment was observed for ATP-dependent enzymes in the molecular function category (Figure 1). These kinds of enzyme activity are involved in multiple biological processes and it is hard to deduce the biological significance of their enrichment in GO analysis.

For type IV genes, translation-related genes were obviously over-represented in the biological process category. Consistently, genes encoding ribosome-localized proteins were clearly over-represented in the cellular component category, and genes encoding ribosome-related proteins were clearly over-represented in the gene function category (Figure 1).

For type I genes, enrichment was observed for oxidoreductase and transferase activity in the molecular function category, but no corresponding enrichment was detected in both biological process category and cellular component category. Some of the stress-responsive genes were identified in type I, but no obviously significant enrichment was detected (Figure S4 in Additional file 3). For type III, genes involved in several important biological processes were represented at similar levels in GO analysis, with no obvious enrichment (Figure S4 in Additional file 3).

### The overall mRNA stabilities for transcripts with different change tendencies during cold stress responses

**Figure 2:**
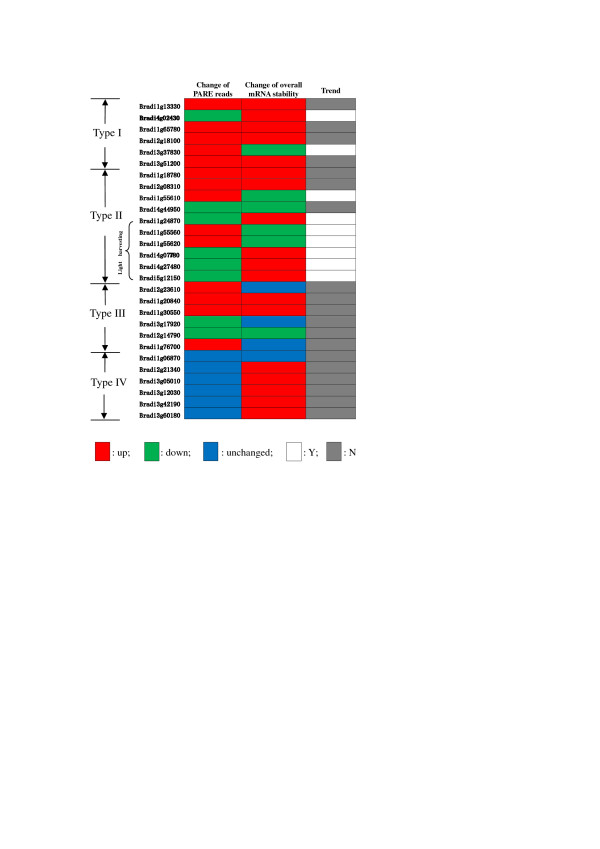
**Relationship between the change of the overall mRNA stability and uncapping-mediated degradation products cold stress response**. The overall mRNA stability was measured through inhibiting transcription by cordycepin. The amount of uncapping-mediated degradation products was indicated by PARE reads. Y: uncapping-mediated degradation products and the overall mRNA stability changed in the opposite direction after cold treatment;N: uncapping-mediated degradation products and the overall mRNA stability changed in the same direction after cold treatment.

To study the effect of cold stress on the overall mRNA stability of type I-IV genes, mRNA half-lives before and after cold treatment were analyzed with cordycepin, a global transcription inhibitor acting as a chain-terminating adenosine analog [39]. Genes randomly selected from type I, III, and IV were analyzed. For type II genes, substantial enrichment was detected for light-harvesting-related genes in the GO analysis. This finding, along with the fundamental role of photosynthesis in plants[40-42], led us to further study this group of genes. Both light-harvesting-related and light-harvesting-unrelated type II genes were analyzed for their overall mRNA stabilities. Interestingly, for light-harvesting-related type II genes, all of the mRNAs with decreased uncapping-mediated degradation products (detected by PARE) showed increased overall stability, and all of the mRNAs with increased uncapping-mediated degradation products (detected by PARE) showed decreased overall stability, implying that uncapping-mediated mRNA degradation may be the key factor in determining the overall mRNA stability for this group of genes (Figure 2, Figure S5 in Additional file 1). For the other groups of genes, their overall mRNA stabilities were either up- or downregulated, or remained unchanged, no clear relationship was detected between the overall mRNA stability and uncapping-mediated mRNA degradation (Figure 2, Figure S5 in Additional file 1).

### Sequence features associated with different mRNA decay patterns

To explore the features of mRNAs with different change patterns, mRNA length, untranslated region (UTR) length, GC content as well as the number of introns were analyzed for type I-IV mRNAs. The connection between introns and mRNA stability has been uncovered recently[43-45]. Interestingly, the average intron number for type II genes was higher than for the other genes, but the percentage of intronless genes was lower in this population (Figure S6 in Additional file 1). These data indicated that a mechanism, more complicated than expected, may exist for uncapping-mediated mRNA stability under stress conditions.

Regulatory sequence motifs in mRNA UTR regions have a close relationship with mRNA stability[46,47]. Genes with the same uncapped/total transcript change patterns, therefore, may share common regulating elements in their mRNA UTR regions. Type I-IV genes were analyzed with an integrated motif-discovery program to search for possible motifs located in 5' or 3'UTRs of corresponding transcripts[48]. Conserved sequence patterns in the 5'UTRs of mRNAs were identified for all four types of genes (Figure S6 in Additional file 1), implying the existing of a specific regulator or regulation mechanism. Interestingly, the identified motifs for type II and type IV genes are similar, providing a clue that similar regulation factor may exist for these two types of genes. The motif-discovery analysis was also performed for the subgroups of type I, II, III, and IV genes, according to the trend of change in PARE and RNA-Seq analysis. More conserved motifs were identified, including several GA-rich motifs (Figure S7 in Additional file 1). It is interesting to note that similar motifs were also found in the 3' UTRs for genes with decreased transcript abundance in both type I and type IV, implying similar expressional regulator for these genes (Figure S7 in Additional file 1).

### Decapping pathway, with clear response to cold stress, makes the most significant contribution to the uncapped transcriptome

**Figure 3:**
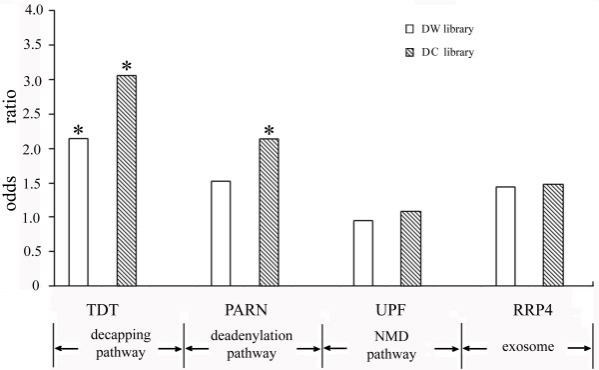
**Enrichment analyses investigating the contribution of different decay pathways to PARE libraries**. The enrichment for targets of mRNA degradation complex core components was analyzed in DW and DC library. Enrichment analyses for target sets, 88 genes for TDT (decapping complex), 135 genes for PARN (deadenylation complex), 58 genes for UPF (NMD complex), and 119 genes for RRP4 (exosome), were performed with Fisher's exact test. Significance of enrichments is showed by two-tailed *P *value. * indicates *P *< 0.001.

A high degree of conservation has been observed for degradation complexes or their core components in plant decay pathways, including the decapping complex, the deadenylation complex, the NMD complex and the exosome[4,5]. Although target sets for the core components of these degradation complexes have been reported in *Arabidopsis*[49-52], such kind of information is hardly available for monocotyledonous plants. To investigate the contribution of different decay pathways to the uncapped transcriptome, enrichment analysis was carried out with target sets in *Arabidopsis*, based on the assumption that degradation complexes have conserved target sets in dicots and monocots (Figure 3, Table S4 in Additional file 2).

Among all the tested mRNA degradation complexes, substrates for the decapping complex showed significant enrichment before and after cold treatment (Figure 3), consistent with the origin of PARE libraries. Significant enrichment was also detected for the substrates of deadenylation complex after cold treatment (Figure 3), suggesting that deadenylation may make more significant contribution to PARE libraries under cold stress condition.

To gain a complete view of the mRNA degradation network's response to cold stress, the targets of the exosome and NMD pathway were also analyzed. No statistically significant enrichment was detected for exosome pathway (Figure 3), which is not unexpected because the exosome mediates the 3'end decay pathway and its degradation products could not be detected by PARE [53,54]. No statistically significant enrichment was detected for targets of the RNA helicase UPF1, the key component of the NMD pathway (Figure 3), implying no direct connection between NMD- and uncapping-mediated degradation.

### miRNA-mediated RNA degradation also makes contribution to the uncapped transcriptome

Endonucleolytic cleavage(s) mediated by miRNA-programmed RNA-induced silencing complexes (RISCs) could also produce 5'-phosphorylated RNAs, which could be detected by PARE [55,56]. In *Brachypodium*, approximately one-fourth of miRNAs showed altered expression (>3-fold) in cold stress. Among them, three conserved (miR169e, miR172b, miR397) and 25 predicted miRNAs respond significantly (≥5-fold) to cold treatment[57]. The target genes for most conserved miRNAs were identified by PARE analysis and most of them were conserved targets (Table 2). The obtained targets were confirmed with biological replicate libraries for DW and DC (Table 2). For the 25 cold-responsive predicted miRNAs, corresponding cleavage remnants for some of them could be found in PARE libraries (Table S5 in Additional file 2).

**Table 2:** The abundance of conserved bdi-miRNAs and their targets before and after cold treatment.

miRNA	Target gene	Function	Ca	miRNAC/W	mRNA C/W	De C/W
bdi-miR156	Bradi4g34667.1	Squamosa promoter binding protein	0	**-**	**-**	**-**
	Bradi3g03510.1	Squamosa promoter binding protein	3	**-**	**↓**	**-**
	Bradi1g26720.1	Squamosa promoter binding protein	3	**-**	**-**	**-**
	Bradi2g59110.1	Squamosa promoter binding protein	0	**-**	**-**	**↓**
bdi-miR160	Bradi3g06487.1	Glucosyltransferase-like protein	0	**-**	**↑**	**↓**
	Bradi1g33160.1	Auxin response factor	0	**-**	**-**	**↑**
	Bradi5g15904.1	Auxin response factor	0	**-**	**-**	**-**
	Bradi3g28950.1	Auxin response factor	0	**-**	**-**	**↑**
	Bradi3g49320.1	Auxin response factor	0	**-**	**-**	**-**
bdi-miR164	Bradi1g32660.1	NAC domain-containing protein	3	**-**	**-**	**-**
	Bradi3g46900.1	NAC domain-containing protein	2	**-**	**↓**	**↑**
	Bradi4g02060.1	NAC domain-containing protein	0	**-**	**-**	**-**
bdi-miR166	Bradi3g28970.1	HD-ZIPIII transcription factor	0	**-**	**-**	**-**
bdi-miR167	Bradi1g32547.1	Auxin response factor	0	**-**	**-**	**-**
bdi-miR168	Bradi1g29577.1	Argonaute 1-like protein	0	**-**	**↓**	**↓**
	Bradi3g05060.1	Hydroxyphenylpyruvatedioxygenase	0	**-**	**-**	**↑**
bdi-miR169	Bradi4g01380.1	Nuclear transcription factor	0	**-**	**↓**	**-**
	Bradi3g57320.1	Nuclear transcription factor	0	**-**	**-**	**-**
	Bradi1g11800.1	Uncharacterized protein	0	**-**	**-**	**-**
	Bradi1g72960.1	Nuclear transcription factor	0	**-**	**↓**	**-**
bdi-miR171	Bradi1g52240.1	Scarecrow-like protein	0	**-**	**↓**	**-**
bdi-miR172	Bradi5g24100.1	APETALA-like protein	0	**↑**	**-**	**-**
	Bradi2g37800.1	APETALA-like protein	0	**↑**	**↓**	**-**
	Bradi1g03880.1	APETALA-like protein	0	**↑**	**↓**	**-**
	Bradi1g30337.1	APETALA-like protein	0	**↑**	**↓**	**-**
bdi-miR393	Bradi5g08680.1	TIR1-like protein	2	**↑**	**↓**	**↑**
	Bradi2g35720.1	TIR1-like protein	0	**↑**	**-**	**↓**
bdi-miR394	Bradi2g59200.1	F-Box protein	0	**-**	**-**	**-**
bdi-miR395	Bradi1g09030.1	ATP-sulfurylase-like protein	0	**-**	**-**	**-**
bdi-miR396	Bradi3g44250.1	Serine/threonine-protein kinase	0	**↑**	**-**	**-**
	Bradi2g11230.1	PTI1-like protein	0	**↑**	**↓**	**-**
	Bradi4g16450.1	Uncharacterized protein	0	**↑**	**↓**	**↑**
	Bradi3g52547.1	Uncharacterized protein	0	**↑**	**↑**	**-**
	Bradi3g57267.1	Uncharacterized protein	0	**↑**	**↓**	**↑**
	Bradi1g12650.1	Uncharacterized protein	0	**↑**	**↓**	**↑**
	Bradi3g51685.1	Uncharacterized protein	0	**↑**	**↓**	**↑**
	Bradi1g50597.1	Uncharacterized protein	0	**↑**	N	**↑**
	Bradi1g09900.1	Uncharacterized protein	0	**↑**	**↓**	**-**
bdi-miR528	Bradi1g24125.1	Uncharacterized protein	0	**-**	**-**	**↑**

N: not detected. Ca: category of miRNA target identification. 0: >1 raw read at the position, abundance at position is equal to the maximum on the transcript, and there is only one maximum on the transcript; 1: >1 raw read at the position, abundance at position is equal to the maximum on the transcript, and there is more than one maximum position on the transcript; 2: >1 raw read at the position, abundance at position is less than the maximum but higher than the median for the transcript; 3: >1 raw read at the position, abundance at position is equal to or less than the median for the transcript; De: PARE reads for miRNA target degradation products. W: sequencing data for non-cold-treated samples. C: sequencing data for cold-treated samples.: no significant changes (log_2 _fold of change was between ±1.5) was identified after cold treatment;: obvious upregulation (log_2 _fold of change >1.5) was identified after cold treatment;: obvious downregulation (log_2 _fold of change < -1.5) was identified after cold treatment. Only conserved miRNAs identified in [57] were involved in the target identification analysis. The miRNA target identification was performed with DW and DC library. The results were confirmed by DW replicate and DC replicate library. To avoid repetition, targets were shown for different members of one miRNA family only when these members exhibited different tend of change in cold stress response. Otherwise, the targets were only shown for the whole miRNA family. No target was detected for miR169e. To reduce the possibility of including false targets in our analysis, miRNA targets not identified in the biological replicate libraries were excluded. The miRNA targets with <5 raw reads at the potential cleavage position were also excluded.

The miRNA libraries, with (WC) and without (NC) cold-treatment, were prepared in the same way as PARE and RNA-Seq libraries[57], enabling direct comparison between different libraries for changes in miRNAs, their target genes, and corresponding degradation products. In *Brachypodium*, miRNAs play different kinds of roles in gene expression regulation. For example, miR393 was a cold-induced miRNA and its target, an F-box gene, was downregulated by cold stress [57](Table 2). Consistently, more miR393 cleavage products were detected by PARE after cold treatment (Table 2), revealing the dominant role of miRNA-mediated regulation. Similar miRNA-target interactions were also observed between miR396 and some of its targets (Table 2). For the other cold-induced conserved miRNAs, miR172, some of their target genes showed obvious decreases in transcript abundance after cold treatment. However, for these target genes, their corresponding degradation products were unchanged in cold stress response (Table 2), suggesting that miRNA-mediated degradation is not the only influencing factor involved in expressional regulation of these genes.

### Endonucleolytic cleavages, another contributing factor to the uncapped transcriptome, were identified and analyzed for its functional mechanism

**Figure 4:**
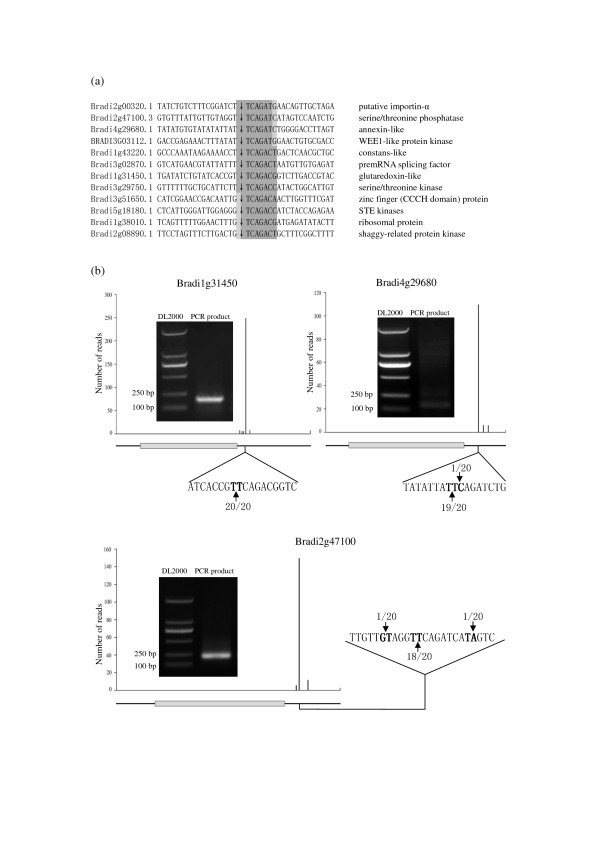
**Examples of endonucleolytic cleavages**.**(a)**A group of genes sharing a motif at the cleavage site which was indicated by'↓'. Those identified endonucleolytic cleavages were also found in corresponding biological replicate PARE libraries. **(b) **PARE reads were shown across the length of mRNAs for endonucleolytic-cleaved genes. Gray bars denoted the coding region of transcripts. The enlarged region showed the sequencing frequencies obtained using RNA Ligase-Mediated (RLM) 5'-RACE assay. The nucleotides in bold letters in the transcripts indicated the cleavages site detected in the PARE analysis. The black arrows indicated digestion sites verified by RLM 5'-RACE with the sequencing frequency (sequencing reads/total sequenced clones) of cloned PCR products. PCR products for the second round of RLM 5' RACE were also indicated, with DL2000 as molecular marker.

In addition to the miRNA-directed internal cleavage pathway, is there any other endogenous cleavage pathway existing in plant? In our PARE analysis, several specific endogenous cleavages were mapped to sites unrecognized by any conserved or predicted miRNAs (Figure 4, Figure S8 in Additional file 1). For all of the transcripts involved in these endogenous cleavage events, PARE reads were identified only at these specific cleavage sites, no reads or reads with obviously lower abundance were identified at other positions (Figure 4b). Moreover, conserved motifs were found at the cleavage sites (Figure 4a, Figure S8 in Additional file 1).The way mRNAs cut in these endogenous cleavage events was quite different from that for miRNA- or siRNA-mediated degradation [55,58], excluding the possibility that they are guided by miRNAs or siRNAs. Plant miRNAs/siRNAs have perfect or near-perfect complementarity to their targets and the cleavage site is located in the central region of the sequence spanned by the guiding small RNAs[55,58]. However, it is not the case for the identified endogenous cleavages, in which the cleavage sites are located at the 5' end of the conserved motifs. For example, 12 mRNAs in the DC library shared a consensus motif, in which '↓' stands for the cleavage site (Figure 4a).

In PARE libraries, approximately 90 transcripts were found to be involved in the identified endogenous cleavage events, when only prominent endogenous cleavages were considered. Ten endogenous cleavage groups were found based on conserved motifs identified at the cleavage sites. Endogenous cleavage groups with fewer than six group members were omitted from further analysis. Interestingly, about four-tenths of the identified endogenous cleavages were located in exons, and approximately six-tenths of them were located in the 3' UTR. Only a few of them were found in the 5' UTR region. These endogenous cleavages distributed randomly in PARE libraries, with no distribution preference identified for any type of genes, implying that this degradation mechanism may be independent of uncapping.

A significant portion of the identified endogenous cleavage groups were cold-stress-responsive. Four groups were identified only in the DW library and four groups were found only in the DC library. These endogenous cleavage groups were also identified in corresponding biological replicate PARE libraries, except for a few genes with relatively low reads (Figure S8 in Additional file 1). Conservation analysis was performed for the conserved motifs identified in these endogenous cleavage groups. The identified motifs expressed different levels of conservation in barley, wheat and rice, but not in *Arabidopsis *(Table S6 in Additional file 2).

No clear functional connection was detected among group members, but a significant portion of them encoded enzymes catalyzing various kinds of biological reactions (Figure S8 in Additional file 1). Of the 12 mRNAs shown in Figure 4, about half of them were enzyme genes (Figure 4). For the DW- or DC-specific endogenous cleavage groups, group members always encoded proteins involved in stress responses. For example, the DC-specific endogenous cleavage group was shown in Figure 4a. Among them, sterile (STE) kinases, serine/threonine kinase, erine/threonine phosphatase, and shaggy-related protein kinase are well known for their roles in stress signal transduction[59-62]. Glutaredoxins belong to glutathione-dependent disulfide oxidoreductases that are involved in oxidative stress responses [63]. Annexins, WEE1-like protein kinase and importin-α have also been reported to be involved in plant stress responses[64-67]. The premRNA splicing factor, ribosomal protein as well as zinc finger protein are implicated in various kinds of cellular processes, including stress responses[68-71].

These observed endogenous cleavages could be made by endonucleolytic enzymes, or be the results of stalled exonuclease activity. If the second case is true, the 5' cap structure and 3' poly(A) tail of their mRNAs would probably be destroyed. To explore the mechanism responsible for these endogenous cleavages, quantitative splinted-ligation reverse transcriptase polymerase chain reaction assay (qSL-RT-PCR) and ligation-mediated poly(A) test (LM-PAT) assay were employed to determine whether these endogenously cleaved mRNA shad intact 5' and 3' end structures, respectively[72,73]. Genes involved in endogenous cleavage events were randomly selected for further analyses. To obtain clear and stable results, genes with low expression levels were not included.

**Figure 5:**
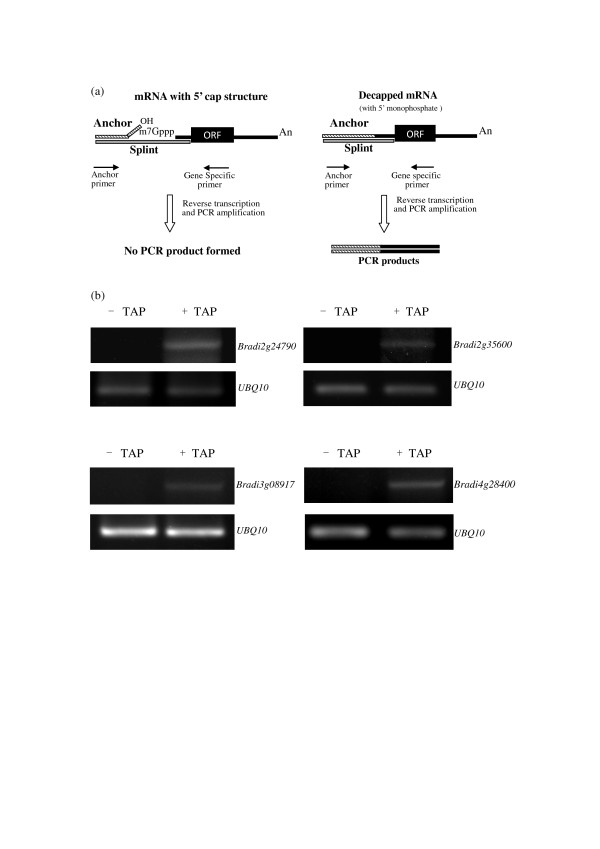
**qSL-RT-PCR assay exploring the 5' cap structure of mRNAs involved in endonucleolytic cleavages in *Brachypodium***. **(a) **Schematic diagram of the qSL-RT-PCR. Solid line: mRNA; Solid rectangle: ORF (open reading frame). The mRNAs containing a 5' cap cannot be ligated to Anchor RNA, but uncapped RNAs, with a 5' monophosphate, can be ligated to the Anchor RNA 3' hydroxyl mediated by complementary DNA Splint oligonucleotides. The ligated RNA is converted to cDNA by reverse transcription with a reverse gene-specific primer. The resulting cDNA is then detected by PCR using gene-specific primer and Anchor primer (a forward primer that anneals to the anchor region). **(b) **Total RNA were treated with (+) or without (-) Tobacco Acid Pyrophosphatase (TAP), an enzyme removing the cap structure from capped mRNA, then annealed with anchor RNA and complementary DNA Splint oligonucleotides. The ligated RNA was converted to cDNA by reverse transcription and the resulting cDNA was detected by PCR, amplified for 30 cycles (+TAP) and 37 cycles (-TAP), respectively. The mRNAs treated with TAP were used as positive control. The *ubiquitin10 *(*UBQ10*) cDNA was used as a control to normalize the amount of cDNA in samples, and 22 cycles were run for *UBQ10 *amplification.

The qSL-RT-PCR assay showed that no RT-PCR products were identified for Bradi2g24790, Bradi2g35600, Bradi3g08917, and Bradi4g28400 (Figure 5), indicating that the anchor RNA could not be ligated to 5'end of these mRNAs because of their intact 5' cap structures. After mRNAs were decapped with Tobacco Acid Pyrophosphatase (TAP), RT-PCR products were detected, suggesting that the qSL-RT-PCR system works well (Figure 5). The results of the qSL-RT-PCR indicated that all the analyzed transcripts had intact 5'cap structures.

**Figure 6:**
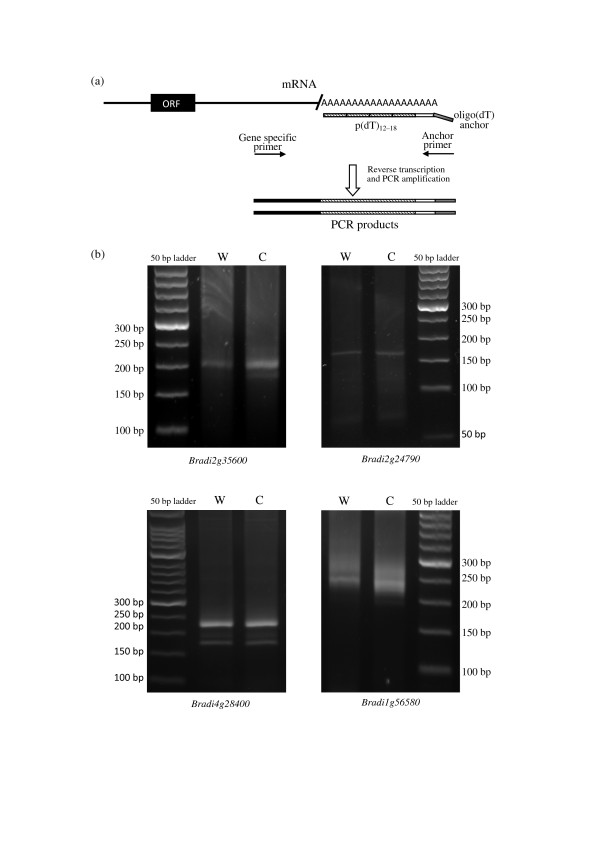
**LM-PAT assay measuring the mRNA poly(A) tail length for genes involved in endonucleolytic cleavages in *Brachypodium***. **(a) **Schematic diagram of the qSL-RT-PCR. Solid line: mRNA; Solid rectangle: ORF (open reading frame). The mRNA is saturated with p(dT)_12-18 _(small rectangles filled with oblique lines)_, _which allows the ligation of adjacent ones. Then, oligo(dT) anchor is ligated to the 3'- most oligo(dT) (small empty rectangle). The synthesis of cDNA from the RNA sample is carried out using the oligo(dT) anchor. PCR is performed with oligo(dT) anchor primer and gene-specific primer. **(b) **Total RNA was extracted from *Brachypodium *seedlings with (C) and without cold treatment (W). Bradi1g56580, an endogenous-cleavage-unrelated type II gene, was used as positive control. PCR products from the LM-PAT assay were analyzed on 2.2% Metaphore agarose gels.

In the LM-PAT assay, poly(A) tail lengths were measured for Bradi2g24790 Bradi2g35600 and Bradi4g28400, which were involved in cold-responsive endogenous cleavage events (Figure S8 in Additional file 1). An endogenous-cleavage-unrelated type II gene, Bradi1g56580, was used as a positive control. As shown in Figure 6, the size of the PCR product for Bradi1g56580 decreased after cold treatment, indicating that the LM-PAT analysis system works. No obvious change was observed in the LM-PAT assay for Bradi2g24790 Bradi2g35600 and Bradi4g28400 (Figure 6), indicating that their mRNA poly(A) tail length did not change after cold treatment. The results of the LM-PAT indicated that although the analyzed transcripts were involved cold-responsive endogenous cleavages, their mRNA poly(A) tail lengths did not respond to cold treatment.

## Discussion

In response to cold stress, eukaryotic cells effectively regulate gene expression through rapidly adjusting their transcriptome[74-76]. Transcript abundance is the equilibrium between the rates of mRNA synthesis and degradation. Up to now, relatively little is known about mRNA degradation. Although the involvement of PBs in stress responses suggested that mRNA decay, especially the decapping pathway, may play a role under stress condition, further experimental evidence is needed in this area. To a certain extent, mRNA degradation is only assumed to be a factor determining the speed by which the transcript abundance is adjusted[77]. Our results indicated that mRNA degradation did play a role in the regulation of steady-state transcript abundance. For example, the opposite change of transcript and uncapped transcript abundance for type III genes uncovered the obvious contribution of uncapping to gene expression regulation (Table 1). Moreover, genome-wide analysis of mRNA degradation under cold stress will deepen our understanding of mRNA degradation and stress response mechanism in plants.

### Different mRNA degradation patterns were uncovered during cold stress responses

Cold stress exerted a clear effect on the uncapped transcriptome. When both gene transcription and transcript degradation were taken into consideration, genes were classified into four groups according to their alternation patterns in response to cold stress (Table 1).

For type I genes, the abundance of transcripts and uncapped transcripts changed in the same direction after cold treatment (Table 1). The mRNA half-life analysis indicated that the overall mRNA stability of type I genes changed during cold stress (Figure 2, Figure S5 in Additional file 1), suggesting that mRNA stability, in addition to transcription, also plays an important role in determining steady-state mRNA abundance. Base on present data, it is not easy to deduce how the uncapping-dependent stability changed for type I genes under cold stress condition.

For type II genes, uncapped transcripts changed significantly after cold treatment, but their transcript abundance remained unchanged (Table 1), suggesting a balance between transcription and transcript degradation. Specific sequence features were identified for type II genes (Figure S6 in Additional file 1), implying a specific regulation mechanism for this group of genes.

For genes belonging to type III, the abundance of their transcripts and uncapped transcripts changed in opposite directions after cold treatment (Table 1). Because the uncapped transcript level is affected by both transcript abundance and its degradation rate, the opposite changes in transcript and uncapped transcript abundance indicate that uncapping did play a role in the regulation of gene expression. For type III genes, no clear relationship was detected between the overall mRNA stability measured with cordycepin-treatment and uncapping-mediated mRNA degradation, implying that uncapping may not be the key factor determining the overall mRNA stability for this group of genes (Figure 2, Figure S5 in Additional file 1).

For type IV genes, their uncapped transcripts remained unchanged after cold treatment although their transcript levels showed obvious alterations (Table 1). For these genes, the opposite changes in transcription and uncapping-mediated degradation reached a balance to ensure that their uncapped transcripts remained stable during cold stress. Now it is not easy to deduce the biological significance of this balance. Translation-related genes were obviously over-represented for type IV genes in GO analysis (Figure 1), implying that some kind of regulation mechanism, preferentially controlling translation, may exist for this group of genes.

The mRNA degradation products from cold-responsive genes account for a proportion of the sequences with high sequencing reads in the DC library (Table S2 in Additional file 2). However, only slight enrichment was observed for stress-related genes in GO analysis (Figure S4 in Additional file 3), implying that the effect of uncapping-mediated degradation cold-stress-related transcripts may be limited to a small group of genes.

### Uncapping plays an important role in gene expression regulation for the light-harvesting process of photosynthesis under cold stress

During cold stress response, the steady-state transcript abundance for type II genes was stable during cold stress, but their uncapping-dependent transcript stability showed significant changes (Table 1). According to GO analysis on type II population, clear over-representation was detected for light harvesting process of photosynthesis (Figure 1). This result, together with the widely accepted important roles of photosynthesis[40-42], led us to further dissect this group of genes. The overall mRNA stability was analyzed for all types of genes, and only light-harvesting-related genes in type II showed opposite alternation trends for uncapping-mediated degradation products and the overall mRNA stability during cold stress response (Figure 2, Figure S5 in Additional file 1), implying that uncapping might be the key factor in determining the overall mRNA stability for this group of genes.

What is the functional significance of this uncapping-mediated regulation mechanism for light-harvesting-related genes during cold stress? Photosynthesis is involved in the first group of biological processes influenced by temperature fluctuation [78,79]. Low temperature causes an energy imbalance in photosynthesis, which results in photoinhibition[78,80]. Annual cold tolerant plants show a decrease in light-saturated rates of CO_2 _uptake in response to a sudden low temperatures stimulus, which is followed by a strong recovery of photosynthesis once their leaves are acclimated [81,82]. For these plants, the photosynthesis activity shows reverse trends of change in short-term and long-term cold responses[78]. One possible explanation of our results is that the expression levels of light-harvesting-related genes remained unchanged to save energy and materials at the middle stage (the stage during which our experiment was performed) of cold response, but their uncapping-dependent transcript stability altered markedly, to prepare for a quick and significant change at gene expression level in case the energy balance in photosynthesis broken and the expression of light-harvesting-related genes needs to be altered instantly under cold stress.

### Decapping, among all the degradation pathways, contributes most significantly to the uncapped transcriptome

In this study, a complete view of the uncapped transcriptome was obtained through PARE analysis. There are different degradation pathways in plants. Up to now, our understanding of them is fairly rudimentary, with several issues remaining to be addressed[3-5]. Based on currently available information, enrichment analysis was carried out and the results indicated that decapping pathway, whose products are 5'-phosphorylated RNAs, is the main contributor to the uncapped polyadenylated degradome (Figure 3).

In addition to the decapping pathway, the NMD and miRNA-directed RISC degradation can also produce 5'-phosphorylated RNAs, which could be identified by PARE. The degradation products for thousands of genes were identified in PARE libraries, and only dozens of them were found to be miRNA targets in our study (Table 2, Table S5 in Additional file 2). To exclude the confusion caused by false miRNA targets, targets with relatively low credibility were not included in our analysis (Table 2). The total number of miRNA targets, therefore, may be underestimated in our study. Up to now, the number of experimental identified miRNAs is limited for monocots[28,83]. However, hundreds or even thousands of miRNA targets have been predicted by software according to the information provided by presently available database for plant miRNA targets[84], suggesting the existence of unidentified miRNA targets. Thus, miRNA-mediated degradation did make contribution to PARE libraries, but presently it is hard to tell its extent when taking the limitation of tools used for miRNA target identification into consideration.

Only limited information is available for NMD pathway in plants [3]. Recent studies showed that both long 3'UTRs and 3' UTR-located introns can efficiently induce NMD in plants and 3'UTR-bound exon junction complex (EJC) is required for intron-based plant NMD[85]. However, the position of NMD in the whole mRNA decay system is still unclear in plant. In the enrichment analysis of PARE library, no statistically significant enrichment was detected for targets of the key component of NMD (Figure 3), providing a clue that NMD may not have close connection with uncapping in plant.

Although the degradation complexes are conserved, they may have different sets of targets in dicots and monocots. Also, it is possible that other undetected degradation complexes may exist in plants. Up to now, relatively little is known about plant degradation pathways. More information is needed for us to better understand their contribution to uncapped transcriptome.

### Endonucleolytic cleavage, identified by PARE, plays a role in the regulation of gene expression, especially under cold stress

The decay of RNA in eukaryotes has long been assumed to be executed mainly by exoribonucleases[86-89]. Recently, however, there are more and more reports demonstrating the involvement of endoribonucleases in RNA turnover [29,90]. In addition to the enzymes displaying endoribonucleolytic activity in general processes associated with RNA metabolism, several endoribonucleases have close connections with specific signals, including stress stimuli[91]. For example, the angiogenin, an RNase A protein with endonucleolytic activity, and endonuclease PMR1 have been proved to be involved in stress response [92-95]. As far as we know, there is no such kind of report in plants. In this study, a series of cold-stress-responsive endogenous cleavage events were detected in PARE libraries, demonstrating the existence of this mechanism in plants. Similar endogenous cleavage events have been reported in mouse[29]. Although PARE analyses have also been performed in plants[25-28], the purpose of those studies is to identify potential miRNA targets, with almost no attention paid on endogenous cleavages.

The mRNAs involved in cold-responsive endogenous cleavage events had intact 5'cap structures and 3'poly(A) tails with unchanged length after cold treatment (Figure 5, Figure 6), suggesting that these endogenous cleavages have no direct connection with decapping and deadenylation. The motifs identified for endogenous cleavage events were not highly conserved (Table S6 in Additional file 2), suggesting that endogenous cleavages may choose different groups of genes as targets in different plant species. The endogenous cleavages distributed randomly among different types of genes. Most of these endonucleolytic cleavages were either cold-induced or cold-suppressed, and their targets were mostly genes involved in cold stress responses (Figure 4, Figure S8 in Additional file 1), suggesting that endonucleolytic cleavage may exert its function in cold stress response. After endonucleolytic cleavage, mRNA degradation can proceed in both 3'-5' and 5'-3' directions, which enabled prompt degradation of the entire transcript. Thus, it is quite possible that the identification of endonucleolytic cleavages uncovers an efficient way to modulate gene expression during cold stress responses.

The mRNA degradation, one of the key parameters determining steady-state gene expression levels, is a complicated and well-ordered process, controlled by multiple mechanisms[96]. Our understanding of mRNA decay system is far from complete, with several pathways remaining unknown. The identification of endonucleolytic cleavages revealed another way of degradation, especially under cold stress condition. With current knowledge of mRNA degradation, the biological significance of this mechanism is still not clear. More efforts are needed in this area to further understand this mechanism and its connection with other degradation pathways.

## Conclusions

Our global analysis provides a complete view of uncapping-mediated mRNA degradation and its related degradation pathways under cold stress. Specific degradation patterns have been uncovered. Endonucleolytic cleavages, another way to modulate gene expression in cold stress response, were revealed. Our results will not only deepen our understanding of mRNA degradation under stress condition, but also help us to gain deeper insight into the cold stress response mechanism of economically important winter-habit crops and biofuel grasses, which has close evolutionary relationship with *Brachypodium*.

## Materials and methods

### Plant material and growth conditions

Seeds of the *Brachypodium distachyon *(L.) Beauv. diploid line ABR5 were placed in petri dishes containing 2 layers of damp sterile filter paper. The seeds were first stratified at 4°C for 1 week to promote synchronous germination, then grown in a growth chamber at 24°C under a 16 h/8 h (light/dark) photoperiod with light intensity of approximately 5,000 lux.

### PARE analysis

Total RNA was extracted from 12-day-old ABR5 seedlings using the mirVana miRNA Isolation Kit (Ambion, Austin, USA) following the manufacturer's protocol. The aerial part of seedlings treated or untreated with cold stress (4°C for 24 h) were pooled and used for construction of the DC or DW library, respectively. PARE libraries were prepared according to [24] in principle. Modification was made according to [97]. Sequencing was performed on Illumina GAIIx by LC Sciences (Houston, TX, USA).

Raw sequencing reads were obtained using Illumina's Pipeline V1.5 software. After removing sequences corresponding to known rRNAs, tRNAs, small nuclear RNAs, small nucleolar RNAs, and repeats/transposons[98,99], the remaining sequences were mapped to the *Brachypodium *genome[33] using bowtie[100], allowing one mismatch. Low quality reads and low frequency reads (≤5) were omitted from further analysis. For PARE reads mapped to multiple positions, reads were divided among different positions. *Brachypodium *draft genome sequences[101] and V1.2 annotation was obtained from [33]. Custom Perl scripts were used to identify the total reads for specific transcripts. For comparison, the DC library was normalized to the DW library based on the population size of the genome-mapped reads (Table S1 in Additional file 2). Genes with log_2 _fold of changes ≥1.5 were considered as upregulated genes and genes with log_2 _fold of changes ≤-1.5 were considered as downregulated genes.

### RNA-Seq

Total RNA was extracted with the mirVana miRNA Isolation Kit (Ambion, Austin, TX, USA) from 12-day-old ABR5 seedlings, which were grown and cold-treated in the same manner as for the PARE libraries. The mRNAs were enriched by using oligo(dT) magnetic beads and broken into short fragments (about 200 bp). The first strand cDNA was synthesized using random hexamer primer with mRNA fragments as templates. DNA polymerase I (Epicentre^® ^Biotechnologies, Madison, WI, USA) was used to synthesize the second strand. The double-strand cDNA was purified with the QiaQuick PCR extraction kit (Qiagen). Then, sequencing adaptors were added and the obtained fragments were purified using agarose gels and enriched by PCR amplification. The resultant products were used for sequencing analysis via Illumina HiSeq™ 2000.

After low quality reads were removed, Illumina sequencing reads were mapped to the *Brachypodium *genome[33] using SOAP[102], allowing two mismatch. Gene expression profiles were obtained with reference to the *Brachypodium *V1.2 annotation[33]. The population size of the genome-mapped unique reads for RC library and RW library was similar, enabling direct comparison. Genes with log_2 _fold of changes ≥1.5 were considered as upregulated genes and genes with log_2 _fold of changes ≤-1.5 were considered as downregulated genes.

### Bioinformatics analysis

All the sequences used for mRNA feature analysis were downloaded from the *Brachypodium *website[33]. As described above, the *Brachypodium *V1.2 annotation was employed in this analysis. Conserved motifs were identified by MEME software [48]. Gene Ontology analysis was performed using agriGO[38]. All other sequence feature analyses were performed using custom scripts in Perl.

For miRNA target identification, PAREsnip[103] was used to detect potentially cleaved targets based on degradome sequences, with *Brachypodium *transcripts (V1.2) and miRNA sequence as input. Conserved miRNA sequences were downloaded from miRBase[104] and *Brachypodium*-specific miRNA sequences were obtained from previous publication [57].

### Enrichment analysis

Enrichment analyses were performed with Fisher's exact test. For the decapping pathway, target sets for TDT (88 genes), the mRNA-decapping enzyme, were analyzed[49]. For the deadenylation pathway, target sets of PARN (135 genes), the poly(A)-specific ribonuclease, were analyzed[50]. For the nonsense-mediated decay (NMD) pathway, target sets of UPF (58 genes), an RNA helicase in the NMD, were analyzed[51]. Target sets of RRP4 (119 genes), a core component of the exosome, were also analyzed[52]. Their presence in the DW and DC libraries was analyzed using the whole *Brachypodium *genome as background. The two-tail *P *value was calculated using online software [105]. Only genes represented in the *Brachypodium *V1.2 annotation [33] were considered in the analysis.

### Total mRNA stability measurement

Total mRNA stability was measured through inhibiting transcription with cordycepin treatment[39,106-108]. The middle part of the primary leaves from 12-day-old *Brachypodium *seedlings with (CD) and without (WT) cold treatment (4°C, 24h) were used. Cordycepin treatment was performed as described[39]. The 3-cm leaf segments were incubated with 600 µM cordycepin solution with regular shaking at 22°C for 7h. Then tissue samples were harvested at regular intervals (1h, 2h, and 4h) and quickly frozen in liquid nitrogen. Total RNA was isolated as described above. Two microgram of total RNA was used for reverse transcription with SuperScript II reverse transcriptase (Invitrogen). The obtained cDNAs were quantified and used as template for quantitative PCR using SYBR Green PCR Master mix (Applied Biosystems, Foster City, CA, USA). For both WT and CD samples, mRNA abundance for the time zero was arbitrarily set to 1 and mRNA abundance for the other time points is shown as the relative value of time zero. Quantitative PCR reactions were repeated three times.

### RLM 5'-RACE

RLM 5'-RACE was conducted using the GeneRacer kit (Invitrogen). Total RNA was isolated as described above. Total RNA was ligated to the GeneRacer RNA Oligo adaptor (5'-CGA CUG GAG CAC GAG GAC ACU GAC AUG GAC UGA AGG AGU AGA AA-3'), then reverse transcription was performed with SuperScript II reverse transcriptase (Invitrogen) using oligo(dT) or gene-specific primers. The obtained cDNAs were subjected to two rounds of PCR amplification (30 to 35 cycles). Primers specific to the RNA Oligo adapter and primers specific to the target genes were designed. For Bradi1g31450,5'-CGA GGA CAC TGA CAT GGA CTG AAG GAG TAG AA-3' (BRADI1G31450-1-F) and 5'-TTG TAG ACA GTG CTA AGC TTC AAG GAC AGG CG-3' (BRADI1G31450-1-R) were used for the first round PCR; BRADI1G31450-1-F and 5'-CGA GCT ATG TCT GAT AGG ACA GAA CAA ATT TCA-3' (BRADI1G31450-2-R) were used for the second round PCR. For Bradi2g47100, 5'-GAG GAC ACT GAC ATG GAC TGA AGG AGT AGA-3' (BRADI2G47100-1-F) and 5'-AGC TGC AAC TCA GAA CGA AAG CTA AAG AG-3' (BRADI2G47100-1-R) were used for the first round PCR; BRADI2G47100-1-F and 5'-AAC AGA GCC AAG CCT GTT TGT GTT GC-3' (BRADI2G47100-2-R) were used for the second round PCR. For Bradi4g29680, 5'-ACT GGA GCA CGA GGA CAC TGA CAT-3' (BRADI4G29680-1-F) and 5'-AAC AGC GTG CAA CCA TTG AAA CCA GGA CAC-3' (BRADI4G29680-1-R) were used for the first round PCR; BRADI4G29680-1-F and 5'-CCG ATT ACA GTC ACC GAA CGA AAT GAC-3' (BRADI4G29680-2-R) were used for the second round PCR. Amplification products from a single band were cloned using the TOPO TA cloning kit (Invitrogen) and sequenced.

### qSL-RT-PCR

qSL-RT-PCR was performed as described previously[72]. Total RNA was extracted as described above and mixed with RNA Oligo adaptor (sequence was provided in RLM 5'-RACE) and complementary DNA Splint oligonucleotides, catalyzed by T4 DNA ligase (Promega, Madison, WI, USA). The mRNAs containing a 5' cap could not be ligated, whereas decapped RNAs with a 5' monophosphate could be ligated with RNA Oligo adaptor. After ligation, the DNA splints were destroyed by RQ1 DNase (Promega).The ligated RNAs were converted to cDNAs by reverse transcription with a reverse gene-specific primer (GSP-R). The resulting cDNAs were then detected by PCR using GSP-R and a forward primer annealing to the RNA Oligo adaptor (adaptor Primer). The reverse transcription and PCR amplification was performed as described above. PCR amplification was run for 30 cycles (+TAP) and 37 cycles (-TAP) for target genes and 22 cycles for *UBQ10*. Primers and DNA Splints used were: for Bradi2g35600, 5'-CGC TGC TAG GGT TTG ACG GTT TTG GTT GGT TTT CTA CTC CTT CAG TCC ATG TCA GTG TCC TCG TGC TCC AGT CG-3' (Bradi2g35600DNAsplint), 5'-GAC TGG AGC ACG AGG ACA CTG ACA T-3' (Bradi2g35600anchor) and5'-GTC GTT GCT GGA CTG GGC CAT CTC TT-3' (Bradi2g35600reverse); for Bradi2g24790, 5'-GCG AGA AGG TGG GGA GGT TTT GGA GGA AGG TTT CTA CTC CTT CAG TCC ATG TCA GTG TCC TCG TGC TCC AGT CG-3' (Bradi2g24790DNAsplint), 5'-GAG GAC ACT GAC ATG GAC TGA AGG AGT AGA-3' (Bradi2g24790anchor) and 5'-ACG AGA GGA AAT AAG GAG ATC CCT CGC T-3' (Bradi2g24790Reverse); for Bradi3g08917, 5'- GAC GGA GCA AGA ACC CGC CGCCGC CAA CCA TTT TCT ACT CCT TCA GTC CAT GTC AGT GTC CTC GTG CTC CAG TCG-3' (Bradi3g08917DNAsplint), 5'-AGG ACA CTG ACA TGG ACT GAA GGA GT-3' (Bradi3g08917anchor) and 5'-TCC AGA GCC AAT GCA TGG ATC AAC AC-3' (Bradi3g08917Reverse); for Bradi4g28400, 5'-GGT TCA GCT CCA ACA ACC TCC ATT CCC ATG TCC CTT TCT ACT CCT TCA GTC CAT GTC AGT GTC CTC GTG CTC CAG TCG-3' (Bradi4g28400DNAsplint), 5'-CTG ACA TGG ACT GAA GGA GTA GAA AGG G-3' (Bradi4g28400anchor) and 5'-CTT AGG GTC CTC AAA TGA TCG GAC CTT G-3'(Bradi4g28400Reverse); for *ubiquitin10 *(*UBQ10*),5'-TCC TCT GAC ACA ATC GAC AAC-3' (UBQ10For) and 5'-TCC TGG ATC TTT GCC TTC AC-3' (UBQ10Rev).

### LM-PAT assay

LM-PAT assays were performed as previously described [73]. The total RNA (250 ng) was incubated with 20 ng phosphorylated oligo(dT)_12-18 _[p(dT)_12-18_] at 65°C for 10 min, then 42°C for 30 min in the presence of T4 DNA ligase (Promega). Then, 200 ng oligo(dT) anchor (5'-GCG AGC TCC GCG GCC GCG TTT TTTTTT TTT-3') was added and the temperature was lowered to 12°C for 2 h so that the oligo(dT) anchor could be annealed to the unpaired ends. This ligated primer was then used for reverse transcription, catalyzed by AMV reverse transcriptase (Promega) at 42°C for 1 h. The first-strand cDNAs were synthesized and used as template for PCR with oligo(dT) anchor primer and gene-specific primer. PCR was performed as described above and primer used was: for Bradi1g56580, 5'-GGC AAT GCG CCA TGT GTG TAT GTA-3'; for Bradi2g24790, 5'-TGG CTC TGC CTT ATT TCA ATT ACT TGA TGC TT-3'; for Bradi2g35600, 5'-CGA GTG TTC GAA ATA AGT AAG CCG TCA GG-3'; forBradi4g28400, 5'-AGT CAA TTC AGC TTG GAT CAG CCC-3'.

### Data availability

Raw data files have been deposited at the National Center for Biotechnology Information Gene Expression Omnibus (NCBI GEO) with accession number GSE48040 and GSE48234.

## Abbreviations

*COR*: cold regulated gene; DC: PARE library to analyze the RNA degradome of *Brachypodium *seedlings with cold treatment; DW: PARE library to analyze the RNA degradome of *Brachypodium *seedlings without cold treatment; GO: Gene Ontology; LM-PAT: ligation-mediated poly(A) test; mRNA: messenger RNA; mRNPs: messenger ribonucleo proteins; ncRNAs: non-coding RNAs; NMD: nonsense-mediated decay; PARE: parallel analysis of RNA ends; PBs: processing bodies; PPDK: pyruvate phosphate dikinase; qSL-RT-PCR: quantitative splinted-ligation reverse transcriptase polymerase chain reaction assay; RNA-Seq: RNA-high-throughput deep sequencing; RC: RNA-Seq library for *Brachypodium *seedlings with cold treatment; RW: RNA-Seq library for *Brachypodium *seedlings without cold treatment; RISCs: RNA-induced silencing complexes; SGs: stress granules; STE kinases: sterile kinases; UTR: untranslated region.

## Authors' contributions

KC and JZ conceived and designed the experiments; JZ performed the experiments; ZM conducted most of the bioinformatic analysis; JZ and KC analyzed the data; JZ and KC wrote the paper. All authors have read and approved the manuscript for publication.

## Supplementary Material

Additional file 1 **Supplemental Figures S1, S2, S3, S5, S6, S7, and corresponding legends**. Figure S1. Cold tolerance and vernalization responsiveness assessment of two *Brachypodium *diploid genotypes, ABR5 and BD21. (a) Seeds for ABR5 and BD21 were germinated and grown for 2 weeks under the growth condition described above. Then the seedlings were treated with -5°C for 6h, 12h, 18h, 24h, and recovered for 10 days. Survival rate was investigated after cold treatment and recovery. Error bars represent standard error. Each time point consisted of 10 to 15 plants, and the experiment was repeated three times.**(b) **Seeds for ABR5 and BD21 were treated with °C for 1, 2, 3, 4, 5, and 6 weeks as vernalization treatment and grown in a growth chamber at 24°C, with 6 h/8 h (light/dark) photoperiod and approximately 5,000 lux light intensity. Flowering time was measured by the number of days after germination until the first flower bud appears. Error bars represent standard error. Each time point consisted of 10 to 15 plants, and the experiment was repeated three times. Figure S2. Reproducibility of PARE libraries. Scatter plot showing the reproducibility between PARE libraries and their biological repeats. The reads from DW **(a) **and DC **(b) **library was mapped to each transcript and the sum of total reads was plotted against corresponding reads from their biological replicate libraries, DW replicate and DC replicate, respectively. Figure S3. Real-time RT-PCR validation of gene expression data provided by RNA-Seq analysis. Total RNA was isolated from 12-day-old *Brachypodium *seedlings with (WC) and without (WO) cold treatment. Real-time PCR analysis was performed as described by Dai et al. (*Plant Physiol *2007, 143:1739). The SuperScripts II reverse transcriptase (Invitrogen, Carlsbad, CA, USA) was used for reverse transcription and SYBR Green PCR Master mix (Applied Biosystems, Foster City, CA, USA) was used for quantitative PCR. The amplification of a *Brachypodium Tubulin *gene was used as an internal control to normalize all data. The normalized mRNA levels in the WO samples were arbitrarily set to 1. Quantitative PCR reactions were repeated three times. Error bars represent the standard error. Figure S5. Overall mRNA stability estimation through quantification of mRNA abundance before and after cordycepin (transcription inhibitor) treatment. The middle part of the primary leaves from 12-day-old *Brachypodium *seedlings with (WC) and without (WO) cold treatment (4°C, 24h) were used. Cordycepin treatment was performed as described (*Plant J *2002, 31:601). The 3-cm leaf segments were incubated with 600µM cordycepin solution with regular shaking at 22°C for 7h. Then tissue samples were harvested at regular intervals (1h, 2h, and 4h) and quickly frozen in liquid nitrogen. Total RNA was isolated with mirVana miRNA Isolation Kit (Ambion, Austin, TX, USA). Two microgram of total RNA was used for reverse transcription with SuperScript II reverse transcriptase (Invitrogen). The obtained cDNAs were quantified and used as template for quantitative PCR using SYBR Green PCR Master mix (Applied Biosystems, Foster City, USA). For both WO and WC samples, mRNA abundance for the time zero was arbitrarily set to 1 and mRNA abundance for the other time points were shown as the relative value of time zero. Quantitative PCR reactions were repeated three times. Error bars indicate standard deviation. Figure S6. mRNA features correlated with different degradation patterns. The mRNA length, UTR length, GC content, and the number of introns were analyzed for type I-IV mRNAs. N: no conserved motif (E-value <0.001) was identified; Y: conserved motifs were identified and the motifs for Y^1^, Y^2^, Y^3^, and Y^4 ^were indicated below the table. The x-axis represented nucleotide position. The y-axis represented the information content measured in bits. The overall height of each stack indicated the sequence conservation at that position, and the height of a letter within the stack indicated the relative frequency of corresponding nucleotide in the motif. Figure S7. Conserved motifs identified in the 5' and 3' UTRs for subgroups of type I, II, III, and IV genes. I, II, III, and IV: different mRNA decay patterns classified according to the variation tend of uncapped transcript/transcript abundance in cold stress. D: uncapped transcript abundance indicated by PARE reads; R: transcript abundance indicated by RNA-Seq reads; N: no conserved motif (E-value <0.001) was identified. Conserved motifs were analyzed with MEME software (*Nucleic Acids Res*. 2006, 34:W369) and identified conserved motifs with E-value <0.001 were shown in the table. Figure S8. Endonucleolytic cleavages identified in the DW and DC library. Transcripts with endonucleolytic cleavages shared different conserved motifs. Conserved motifs, at the endonucleolytic cleavage sites, were marked with gray background. Cleavage sites were indicated by the asterisk. The putative functions of all the transcripts with endonucleolytic cleavages were shown. The majority of the detected endonucleolytic cleavages were also found in corresponding biological replicate PARE libraries. Underlined gene names denoted endonucleolytic cleavages that were not detected in corresponding biological replicate PARE libraries.EW: endonucleolytic cleavage groups only found in the DW library;EC: endonucleolytic cleavage groups only found in the DC library;GONG: endonucleolytic cleavage groups found in both DW and DC library. Click here for file

Additional file 2 **Supplemental Tables S1 to S6 and corresponding captions**. Table S1. Summary of PARE analysis. Table S2. Top 20 genes in DW and DC library. Table S3. Top 10 upregulated and downregulated genes in PARE analysis. Table S4. Enrichment analysis with Fisher's exact test. Table S5. Predicted cold-responsive *Brachypodium *miRNAs and their putative targets. Table S6. The conservation of motifs identified at the endonucleolytic cleavage sites. Click here for file

Additional file 3 **Supplemental Figures S4 and corresponding legend**. Figures S4. Relationship between mRNA decay pattern and gene function. Gene Ontology (GO) analysis was performed for the type I, II, III, and IV genes using agriGO[109] which organizes information for molecular function, biological process, and cellular component categories.type I: (a), molecular function; (b),biological process; (c) cellular component;type II: (d), molecular function; (e),biological process; (f) cellular component;type III: (g), molecular function; (h),biological process; (i) cellular component;type IV: (j), molecular function; (k),biological process; (l) cellular component. Click here for file
