# Supplementary material for: A global profiling of uncapped mRNAs under cold stress reveals specific decay patterns and endonucleolytic cleavages in Brachypodium distachyon
Source: Genome Biol. 2013 Aug 30;14(8):R92. doi: 10.1186/gb-2013-14-8-r92 (PMC4054888; doi:10.1186/gb-2013-14-8-r92)
Supplement: Additional file 1 — Supplemental Figures S1, S2, S3, S5, S6, S7, and corresponding legends. Figure S1. Cold tolerance and vernalization responsiveness assessment of two Brachypodium diploid genotypes, ABR5 and BD21. (a) Seeds for ABR5 and BD21 were germinated and grown for 2 weeks under the growth condition described above. Then the seedlings were treated with -5°C for 6h, 12h, 18h, 24h, and recovered for 10 days. Survival rate was investigated after cold treatment and recovery. Error bars represent standard error. Each time point consisted of 10 to 15 plants, and the experiment was repeated three times.(b) Seeds for ABR5 and BD21 were treated with °C for 1, 2, 3, 4, 5, and 6 weeks as vernalization treatment and grown in a growth chamber at 24°C, with 6 h/8 h (light/dark) photoperiod and approximately 5,000 lux light intensity. Flowering time was measured by the number of days after germination until the first flower bud appears. Error bars represent standard error. Each time point consisted of 10 to 15 plants, and the experiment was repeated three times. Figure S2. Reproducibility of PARE libraries. Scatter plot showing the reproducibility between PARE libraries and their biological repeats. The reads from DW (a) and DC (b) library was mapped to each transcript and the sum of total reads was plotted against corresponding reads from their biological replicate libraries, DW replicate and DC replicate, respectively. Figure S3. Real-time RT-PCR validation of gene expression data provided by RNA-Seq analysis. Total RNA was isolated from 12-day-old Brachypodium seedlings with (WC) and without (WO) cold treatment. Real-time PCR analysis was performed as described by Dai et al. (Plant Physiol 2007, 143:1739). The SuperScripts II reverse transcriptase (Invitrogen, Carlsbad, CA, USA) was used for reverse transcription and SYBR Green PCR Master mix (Applied Biosystems, Foster City, CA, USA) was used for quantitative PCR. The amplification of a Brachypodium Tubulin gene was used as an internal co [file gb-2013-14-8-r92-S1.PDF]

(a)

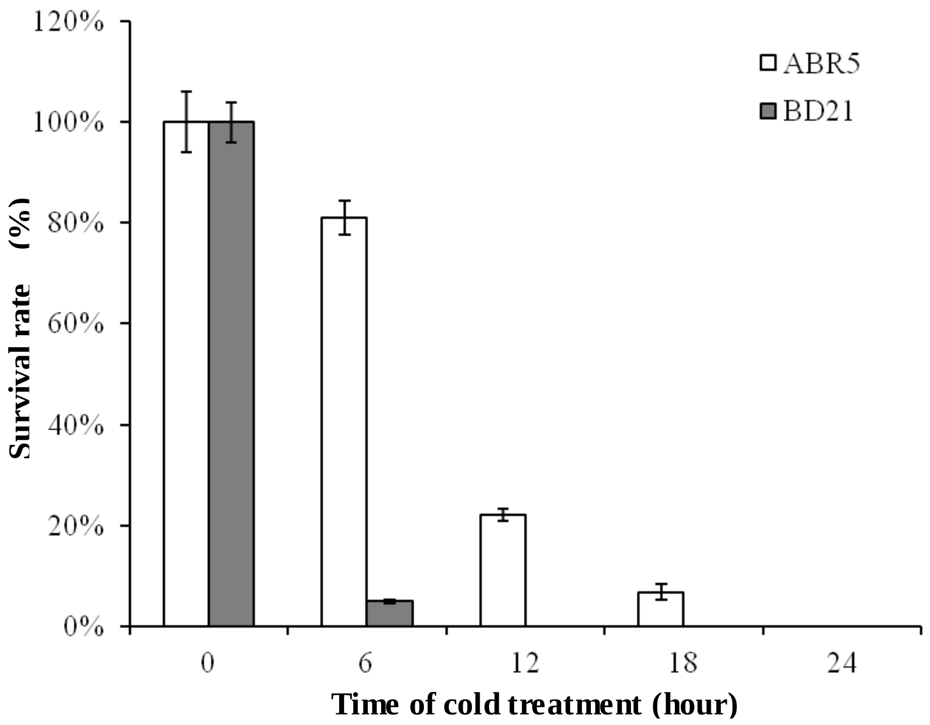

(b)

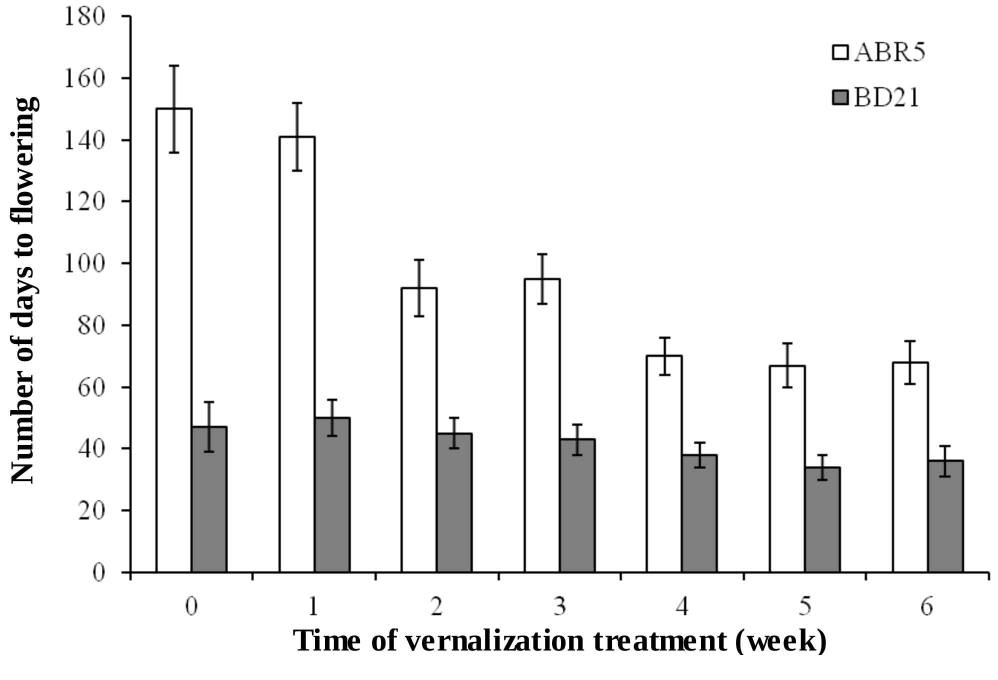

Figure S1. Cold tolerance and vernalization responsiveness assessment of two *Brachypodium* diploid genotypes, ABR5 and BD21.

(a) Seeds for ABR5 and BD21 were germinated and grown for two weeks under the growth condition described above. Then the seedlings were treated with -5℃ for 6h,12h, 18h, 24h and recovered for 10 days. Survival rate was investigated after cold

treatment and recovery. Error bars represent standard error. Each time point consisted of ten to fifteen plants, and the experiment was repeated three times.

(b) Seeds for ABR5 and BD21 were treated with 4 for 1, 2, 3, 4, 5 and 6 weeks as vernalization treatment and grown in a growth chamber at 24 ℃, with 6 hour/8 hour (light/dark) photoperiod and approximately 5,000 lux light intensity. Flowering time was measured by the number of days after germination until the first flower bud appears. Error bars represent standard error. Each time point consisted of ten to fifteen plants, and the experiment was repeated three times.

(a)

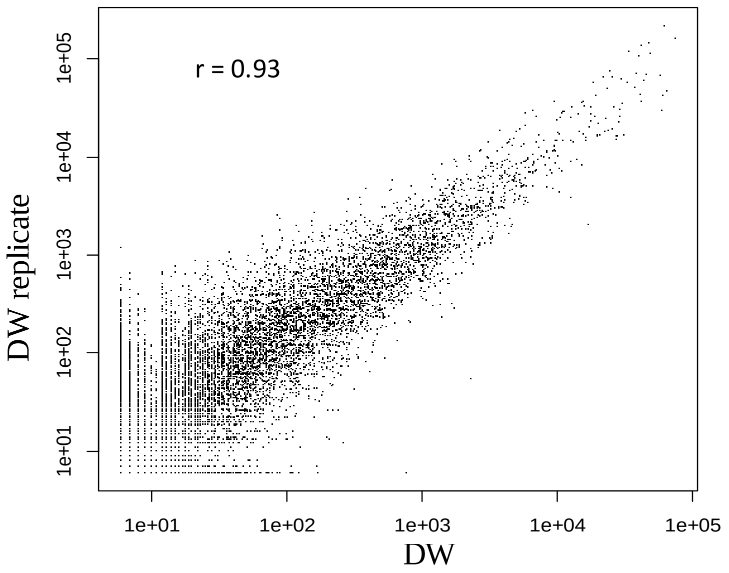

(b)

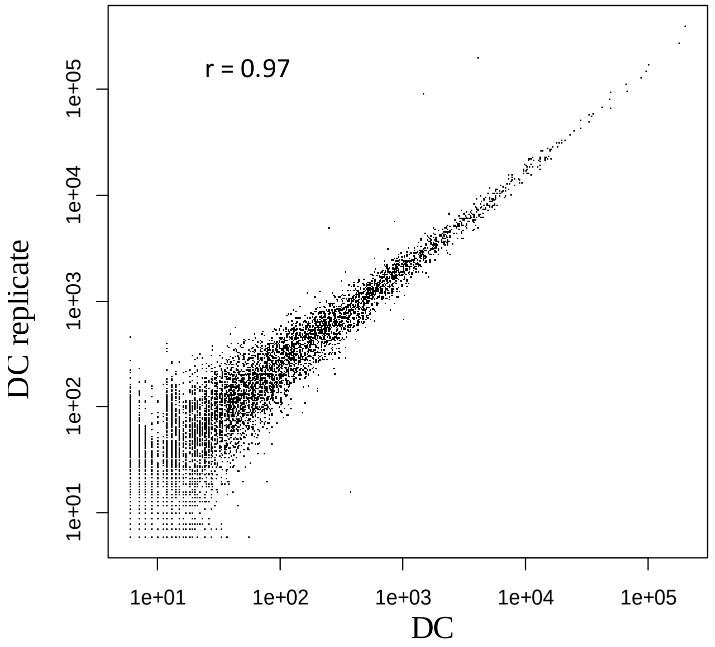

Figure S2. Reproducibility of PARE libraries. Scatter plot showing the reproducibility between PARE libraries and their biological repeats. The reads from DW (a) and DC (b) library was mapped to each transcript and the sum of total reads was plotted against corresponding reads from their biological replicate libraries, DW replicate and DC replicate, respectively.

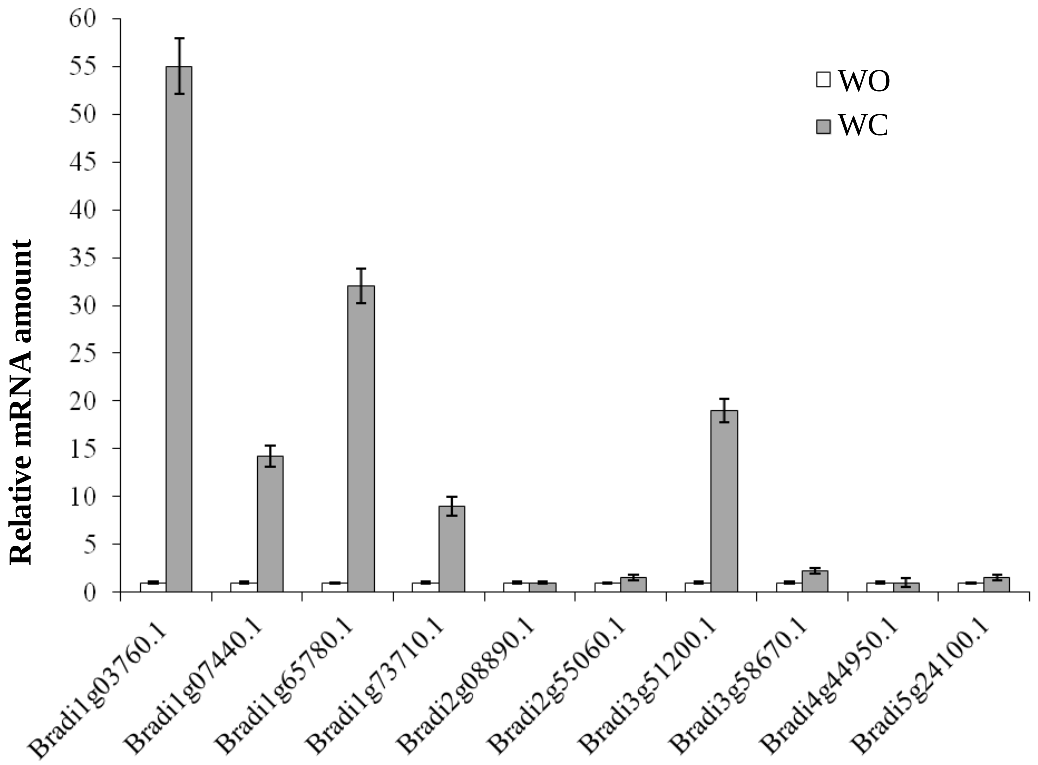

Figure S3. **Real-time RT-PCR validation of gene expression data provided by RNA-Seq analysis.** Total RNA was isolated from 12-day-old *Brachypodium* seedlings with (WC) and without (WO) cold treatment. Real-time PCR analysis was performed as described by Dai *et al.* (Plant Physiol., 2007, 143: 1739). The SuperScripts II reverse transcriptase (Invitrogen, Carlsbad, CA, USA) was used for reverse transcription and SYBR Green PCR Master mix (Applied Biosystems, Foster City, CA, USA) was used for quantitative PCR. The amplification of a *Brachypodium Tubulin* gene was used as an internal control to normalize all data. The normalized mRNA levels in the WO samples were arbitrarily set to 1. Quantitative PCR reactions were repeated three times. Error bars represent the standard error.

The RNA-Seq reads for selected genes:

Bradi5g24100: 19.23 (RW), 36.59 (RC);
Bradi3g58670: 108.11 (RW), 435.76 (RC);
Bradi1g07440: 201.57 (RW), 1540.51 (RC);
Bradi1g65780: 81.55 (RW), 9841.34 (RC);
Bradi2g08890: 61.67 (RW), 100.85 (RC);
Bradi1g03760: 46.55 (RW), 10094.58 (RC);
Bradi3g51200: 15.58 (RW), 4355.57 (RC);
Bradi4g44950: 2.18 (RW), 1.61 (RC);
Bradi2g55060: 0.467 (RW), 1.45 (RC);
Bradi1g73710: 76.92 (RW), 438.45 (RC).

Primers used for real-time RT-PCR were listed as below:

Bradi1g03760Fo: 5’-GTGTGCAACAACAAGTTCCGTGCT-3’;
Bradi1g03760Re: 5’-ACGATCGATCCACGAAGAAAGGCA-3’;

Bradi1g07440.1Fo: 5’-GCCTGGTGTGCTGTTCTCTCTGATAA-3’;
Bradi1g07440.1Re: 5’-CCAATCAGGAAAGTGGCGAGGAAA-3’;

Bradi1g65780.1Fo: 5’-AGTTCCAGGCGCTTCTTCAGT-3’;
Bradi1g65780.1Re: 5’-TATGGTCAGCAGGAGACAGATCCA-3’;

Bradi1g73710.1 Fo: 5’-ACTCCCAGAGTTCGTCAGGAACAA-3’;
Bradi1g73710.1 Re: 5’-TTCGCTCGCAGACAACAGCTACTA-3’.

Bradi2g08890.1 Fo: 5’-GCACACCCATGGCACAAGGTATTT-3’;
Bradi2g08890.1 Re: 5’-AGCAGTGCATCTTAGGTTGGGTGA-3’;

Bradi2g55060.1 Fo: 5’-ATGATCGTCCCACTCGTTCTTGCT-3’;
Bradi2g55060.1 Re: 5’-GATGTTCATGCCGCCCACGTTAAA-3’;

Bradi3g51200.1Fo: 5’-AAGATCAAGGAGAAGCTTCCTGGC-3’;
Bradi3g51200.1Re: 5’-TAATATGCCCTTCTTCTCCGGGCT-3’;

Bradi3g58670.1Fo: 5’- TCAAAGATGCAGAGCAGCTGACGA-3’;
Bradi3g58670.1Re: 5’-TTGTGCGCATGAATGTCGGGAAGT-3’;

Bradi4g44950.1Fo: 5’-ACTGTGGTCTTCTGGAGGACATCT-3’;
Bradi4g44950.1Re: 5’-AAAGGATAGATGCGACAAAGCCCG;

Bradi5g24100.1Fo : 5’-TGGGAGTCGCACATATGGGATTGT-3’;
Bradi5g24100.1Re: 5’-AATGTCGTCGTAATCGTCCAGGCT-3’;

Tubulin:
Forward: 5’-CTCGACAATGAGGCCATCTAT-3’;
Reverse: 5’-TGAGAGACAAGCCTGTTGAG-3’.

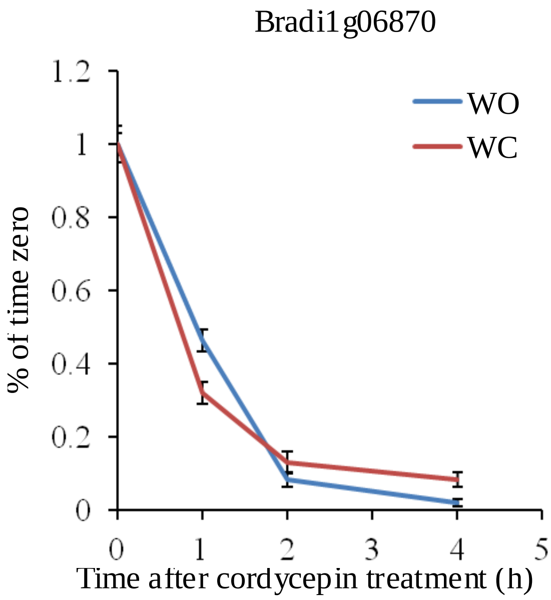
Bradi1g06870
WO
WC
% of time zero
1.2
1
0.8
0.6
0.4
0.2
0
0
1
2
3
4
5
Time after cordycepin treatment (h)

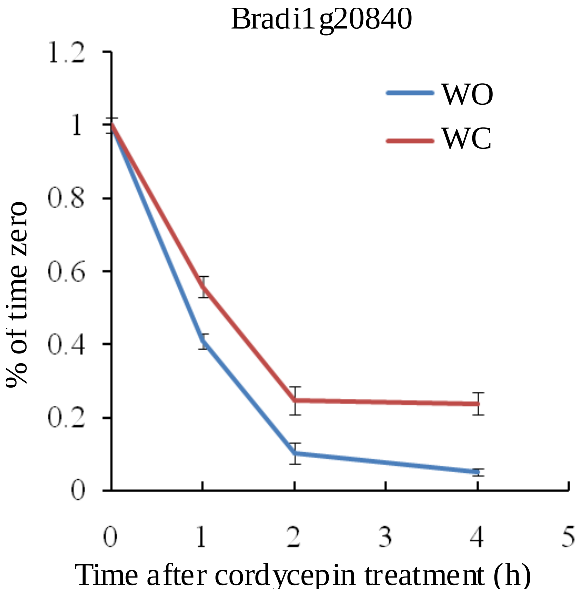
Bradi1g20840
WO
WC
% of time zero
1.2
1
0.8
0.6
0.4
0.2
0
0
1
2
3
4
5
Time after cordycepin treatment (h)

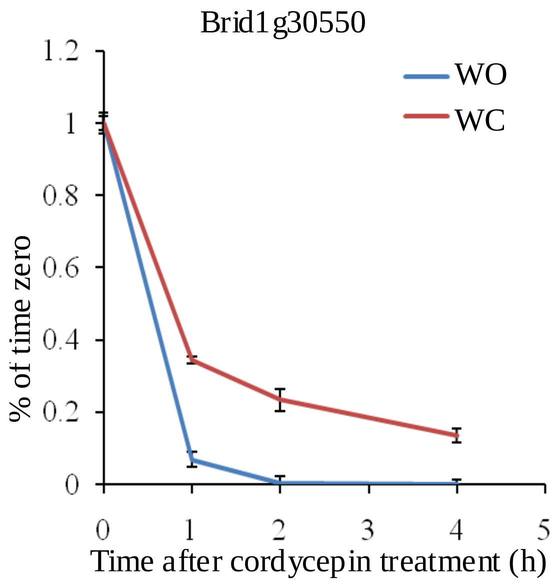
Brid1g30550
WO
WC
% of time zero
1.2
1
0.8
0.6
0.4
0.2
0
0
1
2
3
4
5
Time after cordycepin treatment (h)

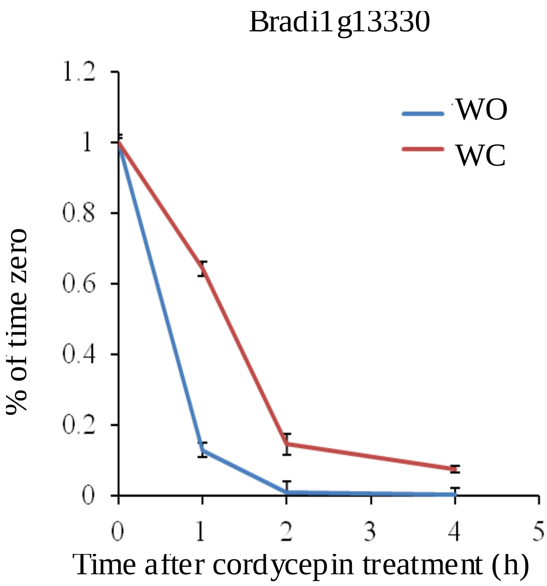
Bradi1g13330
WO
WC
% of time zero
1.2
1
0.8
0.6
0.4
0.2
0
0
1
2
3
4
5
Time after cordycepin treatment (h)

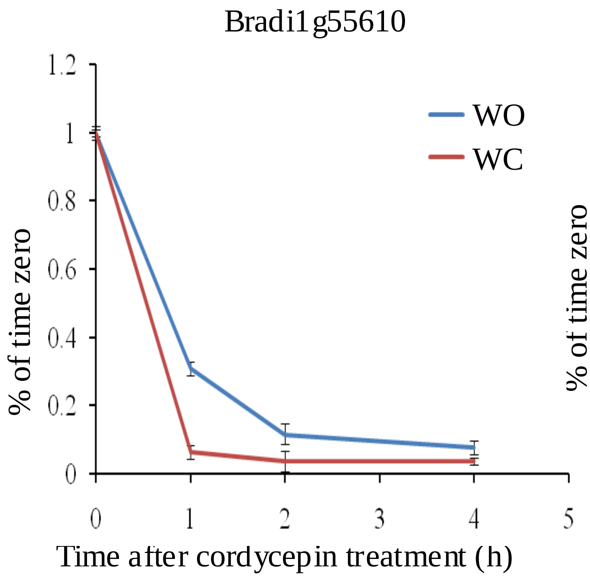
Bradi1g55610
WO
WC
% of time zero
1.2
1
0.8
0.6
0.4
0.2
0
0
1
2
3
4
5
Time after cordycepin treatment (h)

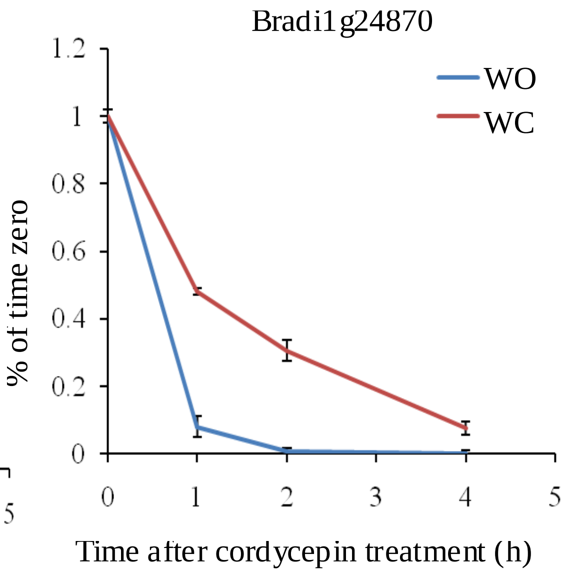
Bradi1g24870
WO
WC
% of time zero
1.2
1
0.8
0.6
0.4
0.2
0
0
1
2
3
4
5
Time after cordycepin treatment (h)

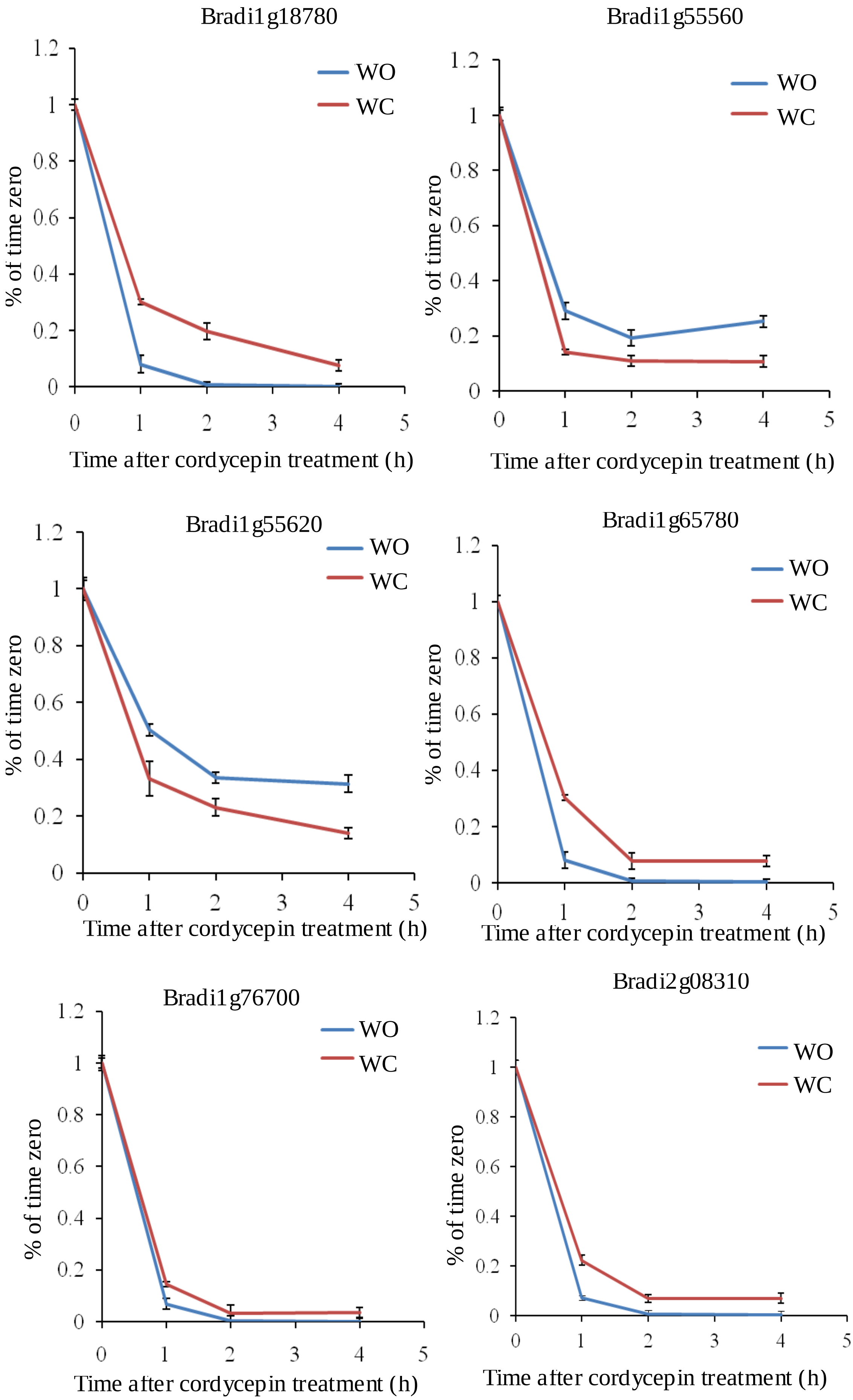
Bradi1g18780
Bradi1g55560
Bradi1g55620
Bradi1g65780
Bradi1g76700
Bradi2g08310
WO
WC
% of time zero
Time after cordycepin treatment (h)
0
0.2
0.4
0.6
0.8
1
1.2
0
1
2
3
4
5

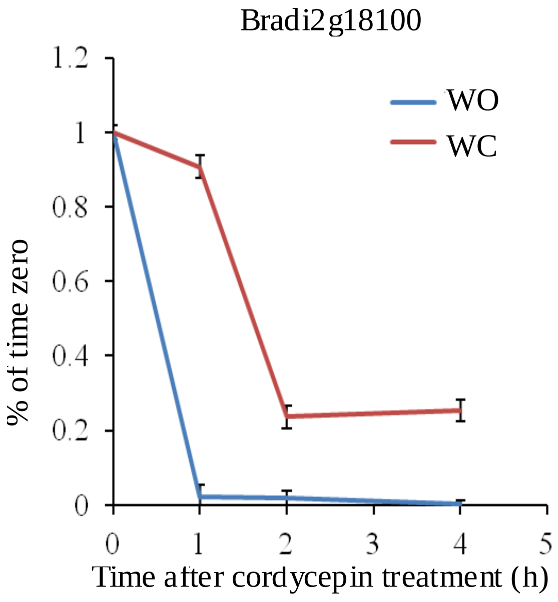
Bradi2g18100
WO
WC
% of time zero
1.2
1
0.8
0.6
0.4
0.2
0
0 1 2 3 4 5
Time after cordycepin treatment (h)

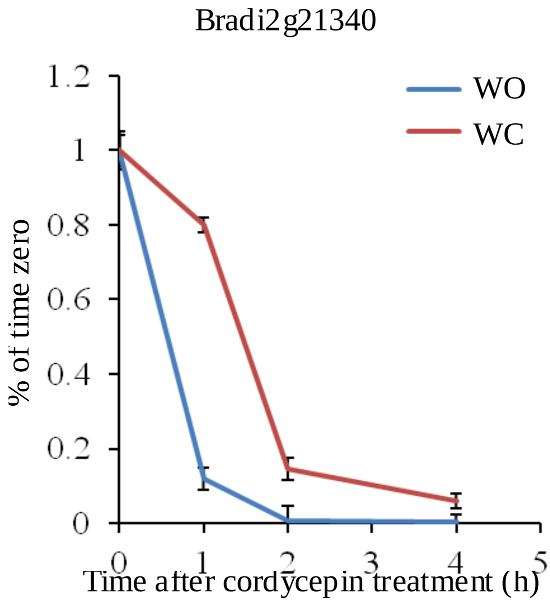
Bradi2g21340
WO
WC
% of time zero
1.2
1
0.8
0.6
0.4
0.2
0
0 1 2 3 4 5
Time after cordycepin treatment (h)

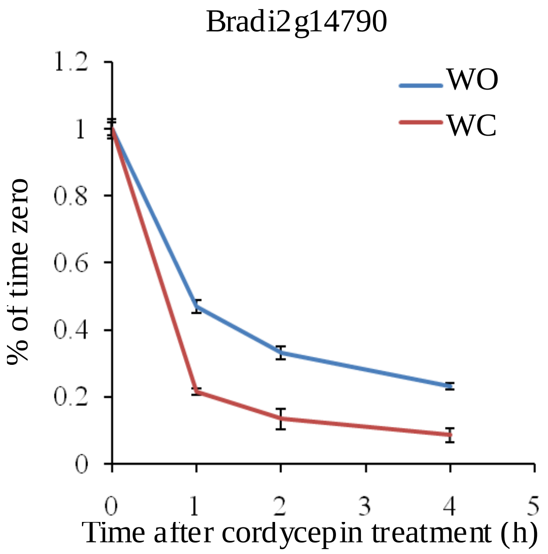
Bradi2g14790
WO
WC
% of time zero
1.2
1
0.8
0.6
0.4
0.2
0
0 1 2 3 4 5
Time after cordycepin treatment (h)

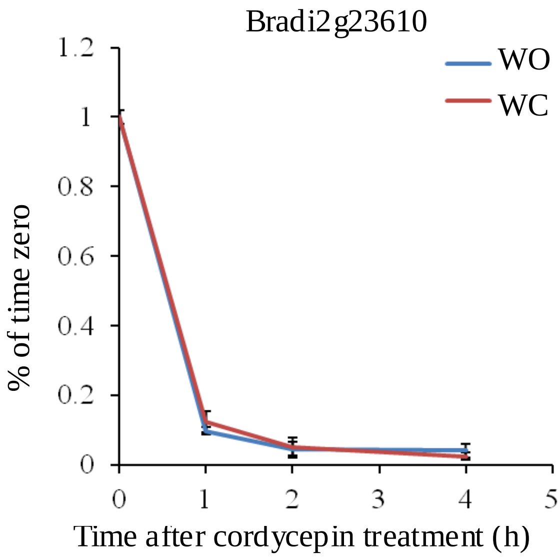
Bradi2g23610
WO
WC
% of time zero
1.2
1
0.8
0.6
0.4
0.2
0
0 1 2 3 4 5
Time after cordycepin treatment (h)

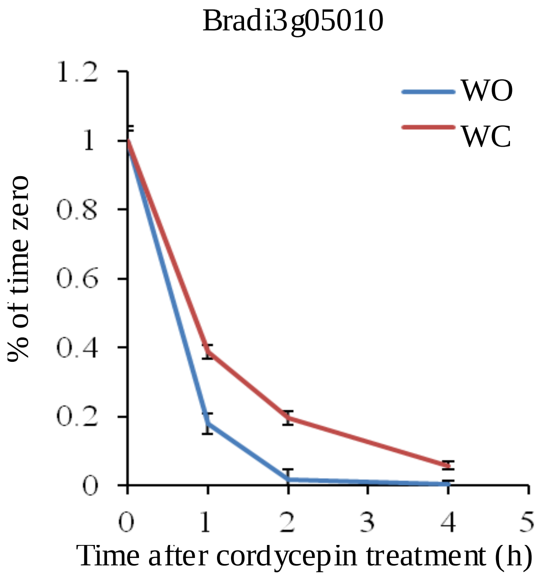
Bradi3g05010
WO
WC
% of time zero
1.2
1
0.8
0.6
0.4
0.2
0
0 1 2 3 4 5
Time after cordycepin treatment (h)

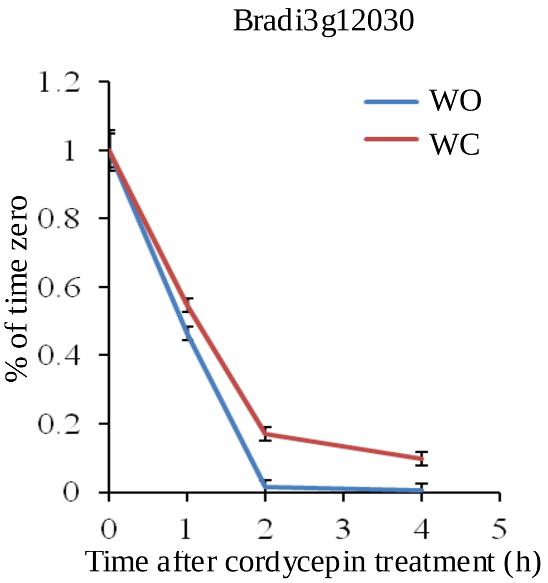
Bradi3g12030
WO
WC
% of time zero
1.2
1
0.8
0.6
0.4
0.2
0
0 1 2 3 4 5
Time after cordycepin treatment (h)

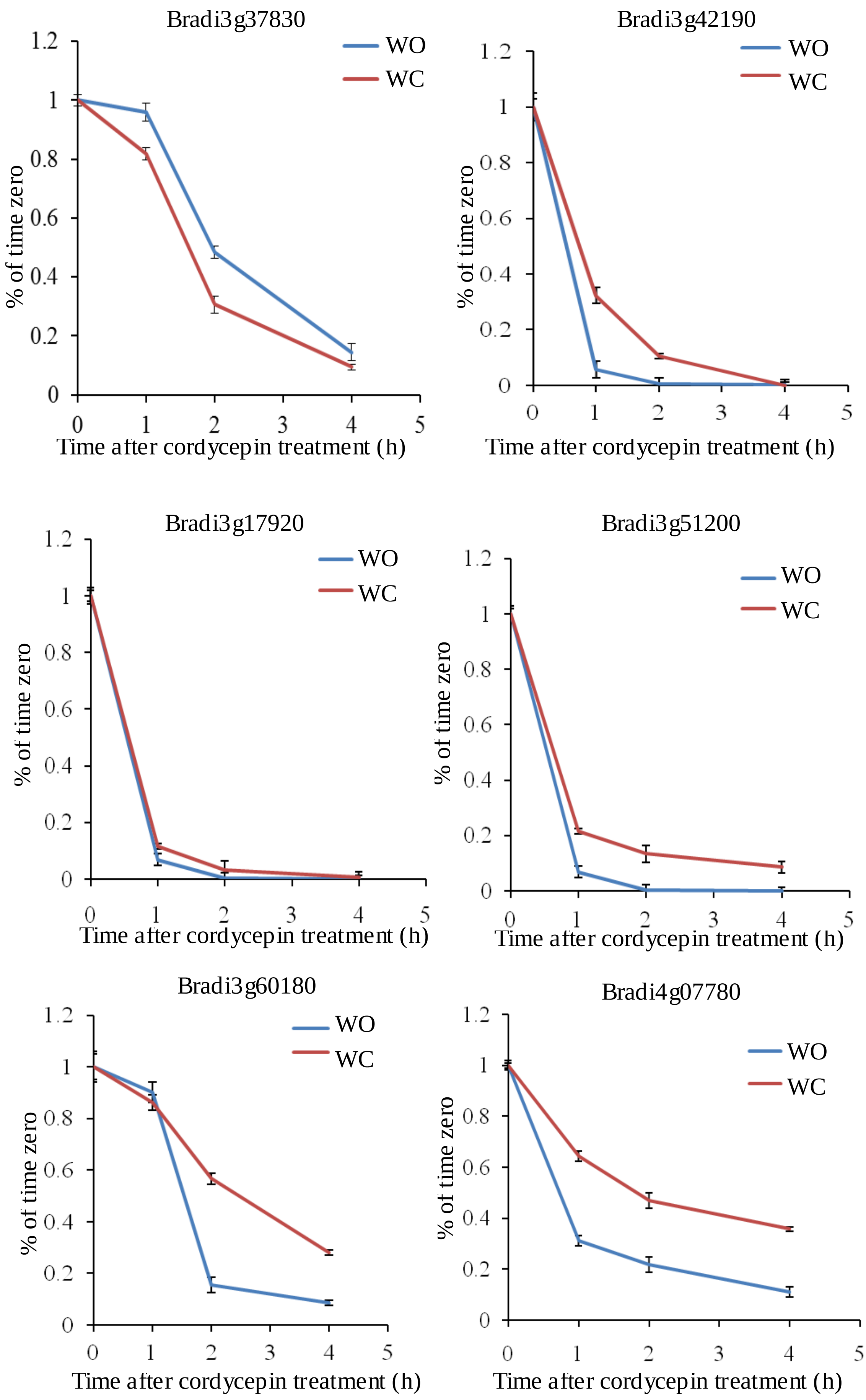
Bradi3g37830
WO
WC
% of time zero
Time after cordycepin treatment (h)
Bradi3g42190
WO
WC
% of time zero
Time after cordycepin treatment (h)
Bradi3g17920
WO
WC
% of time zero
Time after cordycepin treatment (h)
Bradi3g51200
WO
WC
% of time zero
Time after cordycepin treatment (h)
Bradi3g60180
WO
WC
% of time zero
Time after cordycepin treatment (h)
Bradi4g07780
WO
WC
% of time zero
Time after cordycepin treatment (h)

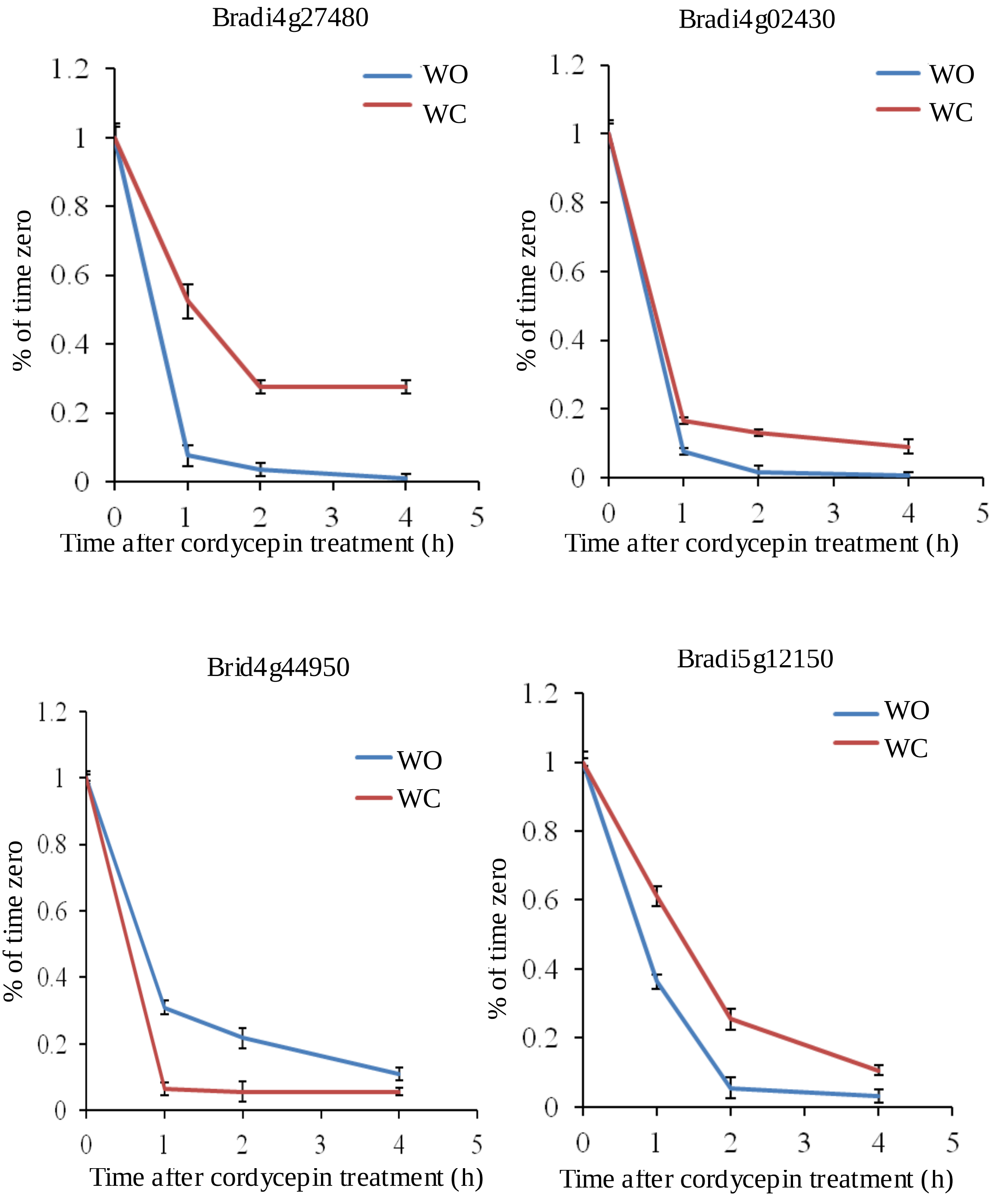

Figure S5. Overall mRNA stability estimation through quantification of mRNA abundance before and after cordycepin (transcription inhibitor) treatment. The middle part of the primaryleaves from 12-day-old *Brachypodium* seedlings with (WC) and without (WO) cold treatment (4℃, 24h) were used. Cordycepin treatment was performed as described (Plant J., 2002, 31:601). The 3-cm leaf segments wereincubated with 600uMcordycepin solution with regular shaking at 22℃ for 7h. Thentissue samples were harvested atregular intervals (1h, 2h and 4h) and quickly frozen in liquid nitrogen. Total RNA was isolated with mirVana miRNA Isolation Kit (Ambion, Austin, TX, USA). Two microgram of total RNA was used for reverse

transcription with SuperScript II reverse transcriptase (Invitrogen).The obtained cDNAs were quantified and used as template for quantitative PCR using SYBR Green PCR Master mix (Applied Biosystems, Foster City, USA).For both WC and WO samples, mRNA abundance for the time zero was arbitrarily set to 1 and mRNA abundance for the other time points were shown as the relative value of time zero. Quantitative PCR reactions were repeated three times. Error bars indicate standard deviation.

Primers used for real-time RT-PCR were listed as below:

Sta1G06870For:
5’-AGCCATGGGTAAGGAGAAGACTCA-3’;
Sta1G06870Rev:
5’-AAGCTTGTAGATCAGGTGGCCAGT-3’;

Sta1g18780For:
5’-GGTGGGAACTCGTCAACTC-3’;
Sta1g18780Rev:
5’-GTCGTGCTCGTCTTCTTCTAC-3’;

Stag1g13330 For:
5’-AGCAAAGATCACCACAGCGCATTC-3’;
Sta1g13330 Rev:
5’-CCATGGCCGGAGTTGTTTCTTCTT-3’;

Sta1g20840For:
5’- GTGGTGGTGACCTGCATATT-3’;
Sta1g20840Rev:
5’-CTGCTGGATGGTTAGCTTAGAG -3’;

Sta1G24870For:
5’-TCGAGTGAGTACGTATAGCAGCCA-3’;
Sta1G24870Rev:
5’-TTCTTCGCCGGCGCCTTCTT-3’;

Sta1g30550For:
5’-GGCTTTAGAGGCAGAAGAACA -3’;
Sta1g30550Rev:
5’-GGAAACAGATGAGGGAGCATAA -3’;

Sta1g55610For:
5’-CCCACGCACAACATCCTT -3’;
Sta1g55610Rev:

5’-AAGGACAGGATGTCCCAGAT-3’;

Sta1G55560For:
5’-GCCAAGTGCGATCCATCTAGTAGCTT-3’;
Sta1G55560Rev:
5’-AAAGCTAGTGCACGACGGAGCAAA-3’;

Sta1G55620For:
5’-ACCATGATGGCCTTGAGCTCCTT-3’;
Sta1G55620Rev:
5’-TCGTCTCCTTGGTCGGGTCCATTT-3’;

Sta1g65780 For:
5’-TGCAGTTCCAGGCGCTTCTTCAGT-3’;
Sta1g65780 Rev:
5’-ATGATGGCGAGGATCACCTCCAGGAA-3’;

Sta1g76700For:
5’-AACGGCGCAAGGGAAATA-3’;
Sta1g76700Rev:
5’-GCGCATCCTTCATTTGGTAATC -3’;

Sta2g08310For:
5’-AACGCCATCATCGTCAACACAGCA-3’;
Sta2g08310Rev:
5’-TCGAGCCACTTAACACACGCATCT-3’;

Sta2g14790For:
5’- TACTCGAGACGCTGTACCA-3’;
Sta2g14790Rev:
5’-CTTCTTCTCCGCCTCCTTTATC -3’;

Sta2g18100For:
5’-TCCAACACGAACCAGGCGAGCTA-3’;
Sta2g18100Rev:
5’-TTGTCCTTGGTCGCGCCCATCAT-3’;

Sta2G21340For:
5’-GCTCCCTCCGTTCGTCGAATTTAAGA-3’;
Sta2G21340Rev:
5’-ATGCCATGCTCATCACACACAACC-3’;

Sta2g23610For:
5’-CATGGGTTTGAAGAACAGGATTG-3’;
Sta2g23610Rev:
5’-CAGAAGGGCAGGCCTAATAAA-3’;

Sta2g40870For :
5’-GCACACCGTCTTCCCAGCCATAAA-3’;
Sta2g40870Rev :
5’-TCTTCTCTTCTTTGGGTGCTGCTG-3’;

Sta3G05010For:
5’-AAATCAAATCGGCGCCTCCACACT-3’;
Sta3G05010Rev:
5’-TCGCAGATCACCTCCCAGAACTT-3’;

Sta3G12030For:
5’-CAGGCCAATACATATCACAGGCGAGA-3’;
Sta3G12030Rev:
5’-ATCTCTGCGTCGTGTTCCATCTCT-3’;

Sta3g37830For:
5’-TTTCCTTCCTCCCACAGTAGCAGC-3’;
Sta3g37830Rev :
5’-ATGATCTGGTACGCCGCCTCCTTT-3’;

Sta3G42190For:
5’-TACACCTACGACAGGGTGTTTGGC-3’;
Sta3G42190Rev:
5’-TTCCACTGACGACTGATAGAGCGA-3’;

Sta3g51200For:
5’-GCCACAAGCCAAGAACCAACCAAT-3’;
Sta3g51200Rev:
5’-TGCTCCTCTCATCCTCCATGATCG-3’;

Sta3g17920For:
5’-GCTGGTCTCTGCTTACAAACTA-3’;
Sta3g17920Rev:
5’-GCGTTCTAGACACAACCACA-3’;

Sta3G60180For:
5’-TCCCTCATCGCAGCTCTCTCT-3’;

Sta3G60180Rev:
5’-ACTGGTTCGACTTGGACCTCTTGT-3’;

Sta4g02430For:
5’-CCCGACAACCCAACTCTATAC-3’;
Sta4g02430Rev:
5’-CCATTGTGGCTTGAGCTTTAC-3’;

Sta4G07380 For:
5’-ATCTTGTCCGGATACACCGATGCT-3’;
Sta4G07380Rev:
5’-TGACGATGGCAAGTTCTTTACGGC-3’;

Sta4G27480For:
5’-AGTGCTGGTGTTCTCCGCC-3’;
Sta4G27480Rev:
5’-GCTGAGGCCTTCCTGTCCT-3’;

Sta4g44950For:
5’-GAATCAGGACAAGCGTTGGTGGAT-3’;
Sta4g44950Rev:
5’-AGCACTTATGTGCACCAGCTTCCT-3’;

Sta5g12150For:
5’-AACTCCACGGGCGATCAAGGATA-3’;
Sta5g12150Rev:
5’-AGTAGAAGATGAGCATGGCGAGCA-3’;

Figure S6. mRNA features correlated with different degradation patterns.

Type		Motif	Average UTR length	Average total length	Average number of introns in UTR	Average number of introns	Percentage of intronless gene (%)	GC content in UTR (%)	GC content of mRNA (%)
I	5'-UTR	**Y^1**	158	1641	0.25	4.63	14.29	0.55	0.53
	3'-UTR	N	349		0.10			0.41	
II	5'-UTR	**Y^2**	172	1890	0.30	6.56	9.43	0.57	0.51
	3'-UTR	N	387		0.15			0.41	
III	5'-UTR	**Y^3**	140	1606	0.22	4.44	17.50	0.56	0.54
	3'-UTR	N	350		0.09			0.41	
IV	5'-UTR	**Y^4**	137	1653	0.19	5.43	10.10	0.58	0.52
	3'-UTR	N	388		0.07			0.42	

Y^1:

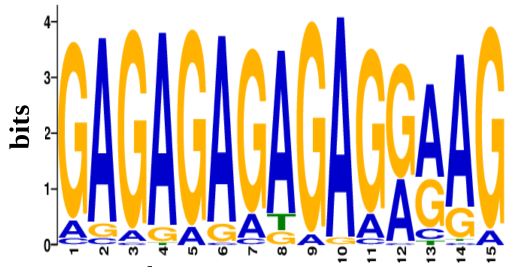

E-value: 4.5e-025

Y^2:

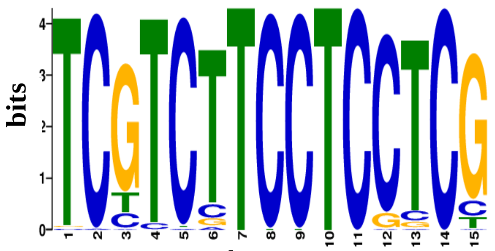

E-value : 6.5e-064

Y^3:

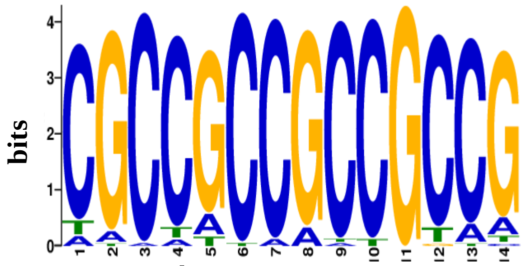

E-value: 3.6e-020

Y^4:

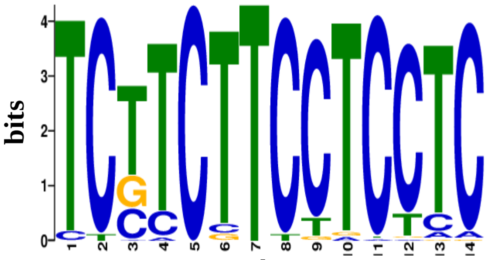

E-value : 2.3e-019

The mRNA length, UTR length, GC content and the number of introns were analyzed for type I-IV mRNAs. N: no conserved motif (E-value < 0.001) was identified; Y: conserved motifs were identified and the motifs for Y^1, Y^2, Y^3 and Y^4 were indicated below the table. The x-axis represented nucleotide position. The y-axis represented the information content measured in bits. The overall height of each stack indicated the sequence conservation at that position, and the height of a letter within the stack indicated the relative frequency of corresponding nucleotide in the motif.

Figure S7. Conserved motifs identified in the 5' and 3' UTRs for subgroups of type I, II, III and IV genes.

Type	Change pattern		Identified conserved motif in 5' UTR	Identified conserved motif in 3' UTR
	D	R		
I	↑	↑	N	N
	↓	↓	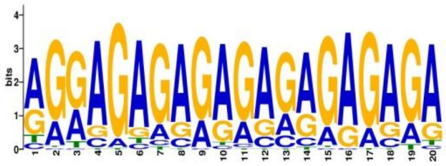 E-value: 5.6e-006	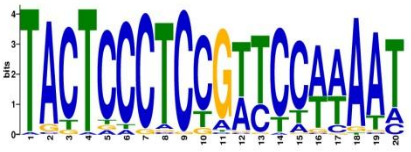 E-value: 1.3e-024
II	↑	—	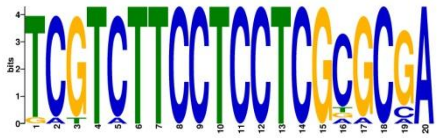 E-value: 2.1e-024	N
	↓	—	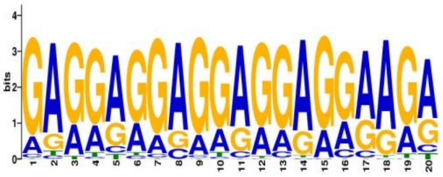 E-value: 1.9e-044	N
III	↑	↓	N	N
	↓	↑	N	N
IV	—	↑	N	N
	—	↓	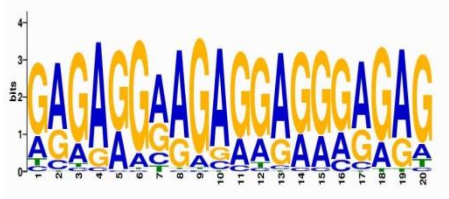 E-value: 1.4e-017	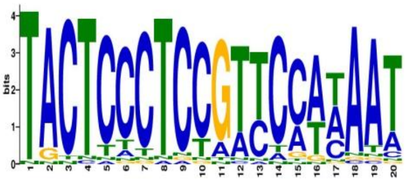 E-value: 1.1e-039

I, II, III and IV: different mRNA decay patterns classified according to the variation tend of uncapped transcript/transcript abundance in cold stress. D: uncapped transcript

abundance indicated by PARE reads; R: transcript abundance indicated by RNA-Seq reads; N: no conserved motif (E-value < 0.001) was identified. Conserved motifs were analyzed with MEME software (Nucleic Acids Research, 2006, 34:W369) and identified conserved motifs with E-value < 0.001 were shown in the table.

GONG1

BRADI1G31390.1	TAATTAAAAGAGAAACG*AGAGAGACAGAGAGCAAAAGAAGGGT	SPL10
BRADI2G03350.1	ACAACTTTGTTCTTGGG*AGAGAGAGCATATGGTATGCAACCGT	ALBINO3
BRADI3G29760.1	AATTCTGACAAGCAGAG*AGAGAGAGCAATGTAATTTGAACTGT	Unknown protein
BRADI5G22547.1	GTCAGCTTGGGCCACTG*AGAGAGATTGGAGGATCACTTCACAT	Unknown protein
BRADI1G63610.1	GAGTCCTGCTTTCTGTG*AGAGAAAGGAGAGAGGAGTTGTGTGT	Unknown protein
BRADI4G00450.1	GCCGACCCGTCCAGCAG*AGAGGAGCAAGTCCAAGCGAAGAGGG	HTRA3
BRADI1G30648.1	TGCTGCAGCACATATTG*AGAGAGAGTTGCAAATCACAAGCTGG	TFIID

GONG2

BRADI1G71450.3	GCCAAGGCAGCAG*TTGGATCACGGAGAGAGCCTCAAGTT	unknown protein
BRADI1G37790.1	CTTAATGCTATGG*TTGGACCTTGTTTCATATCTGCTTTG	ATPase
BRADI3G00330.1	AACAAGATCGAAT*TTGGACATGGAGCAAGTTTTATCAGT	glyoxylate reductase
BRADI2G26547.1	GCAGTGTACAGGT*TTGGACCTGAACATTAATTTAATTCA	protein ligase
BRADI3G38390.1	CAAATCGGCTGCT*TTGGACCTCACTGTTTTGGAGGCTGT	ATPase
BRADI4G43910.1	GCTTGTGTTTGGT*TTGGAAACTCAAGTCTTTGAGCTGTT	GTPase
BRADI3G34800.1	AATGAACCCATAA*TTGGACAACAGAATCAGTTTACTATA	ATPase

EW1

BRADI1G13930.1	GCGGCTTATCAACGAGGAG*TACAAGATCTGGAAGAAGAACAC	p55-like protein
BRADI2G35767.1	GTAATCGAAGAGAGGATTT*TACAACTTATCTTATGTACAATG	RGA4-like protein
BRADI3G17550.1	TATGTAATAACTGTTGTGT*TACACTGCAAGATGCATCGAGTT	dehydrogenase
BRADI4G26487.2	CTTTCGAACAGAGTTAAAT*TACACCTTTAGATTGTATTGGTT	ndopeptidase
BRADI1G33970.1	ACCGCAGCACCTTTACTGA*TACACTGGAATCAAATAAACCAT	Unknown protein
BRADI5G12227.1	AGTGTATTGTACGTGCACA*TACACTTGATCGGTGATAATGCG	receptor kinase
BRADI5G24160.1	TTCTTTTCCGTTTGTGCTC*TACACTATACTATAGCCATACAT	ATPase
BRADI2G35600.1	GCCAAAGGCCCGTGACCTC*TACACTCGCTACGAGCCCGTGGC	REF/SRPP-like protein
BRADI2G11830.1	AAGGGAAAAGTCGGAGCGC*TACAGTTGTCCTTGCCTACCTGA	phosphatase

EW2

BRADI3G08917.1	TGATTTGAGTTATGT*TCAAACAAGCTGTGAACTTGA	protein kinase
BRADI2G56120.2	CTAGATATTCAGTAT*TCAAACCTCAGTGAATTGCAA	polypyrimidine-binding protein
BRADI2G46550.1	CAACAACCTACATCT*TCAAGGAGAAGAGCCCCAAAA	RNA polymerase
BRADI2G13090.1	GCTTGGGCAACGAAA*TCAAGTCCTGTTCCCCTTTTT	ATPase
BRADI4G28400.1	ACACCCTCTACTTCA*TCAAGGTCCGATCATTTGAGG	unknown protein
BRADI1G51530.1	TTATGCCTTCTCGGA*TCAAGAAAGAGGGGTCCACTG	protease like protein
BRADI1G26210.1	CGGGGTGCTAAATTA*TCAAACTAGTTCATGACTGAA	RNA binding protein
BRADI3G27327.1	AAGGCGATTTCAGAA*TCAAAAAAAGTAAAGTCAAAA	Ycf4

EW3

BRADI2G21340.1	CGAGCTTACCCAGCAG*ATGTGGGATTCCAAGAACATGATGT	GTPase
BRADI1G32430.1	AGAGCTGACTCAGCAG*ATGTGGGACTCAAAGAACATGATGT	GTPase
BRADI2G56000.1	CAGTACCTTCCAGCAG*ATGTGGATCTCCAAGGCAGAGTACG	GTPase
BRADI1G38400.1	TCACGACGTCCAGCAG*ATGTAGGGAGCTTCAATCTCCTTCC	Unknown protein
BRADI3G26880.1	AAAGACTTCGCAGCAG*ATGTAATCGTGATGTGGAACTGCAA	GTPase
BRADI1G06860.8	TGAGACTCTTGAAACT*ATGTAAGCAATGTTCATGTCTGTCG	GTPase
BRADI3G52437.1	GTAGTATGGTAAATCT*ATGTACAGTCAGTAACTGACAGTTC	pyrophosphohydrolase

EW4

BRADI3G53040.1	ATATATGTACCTTAA*TGGATCTCTAGGCTCTAGCTGACAA	Rab GTPase
BRADI1G06750.1	CAGATCTGTATTTAC*TGGATCAACCTACCACCGTGAAAAC	phosphate isomerase
BRADI1G35900.1	AATGTTTATTCTTAC*TGGATTGCAGACTACCTTCTGCTTG	unknown protein
BRADI2G37460.1	CCCTTCACATGTATC*TGGATCAATATTTGCAACTGGCCAA	glycosyl hydrolase
BRADI1G13910.1	CTGTTAATGGATTTC*TGGATGATGGCTGGTCATTGATGAG	HD-ZIP III
BRADI1G02310.1	CCTCTTGGTTGGTAC*TGGATCGTGTTCGGCCCCTGTTTCCA	FGFR1
BRADI2G09550.1	ACCATAAATTGGATC*TGGACACTGAAGGCCTGAAGCTCTG	epimerase
BRADI2G08287.1	TTATCCCTGTATCAC*TGGACTGGTAAATGAATAACATGCA	GLK2
BRADI1G75750.1	GGTTGGTGAGGCTTC*TGGACAAGATGGATATACAGCAGCG	U-box protein
BRADI1G54400.1	ACCTCTTGGCGTATC*TGGACAGCATACTATCTGGTCTCTG	MAF1
BRADI2G43650.1	GAGTCAGGATAATCC*TGGACATATATATTGTATTTCGGTC	peptide transferase
BRADI4G25177.1	TATATAGTTTGTAGC*TGGACATGAATTATGTAGGTTTTGG	ubiquitin protein ligase
BRADI5G21650.1	TTATCCCTGTATTAC*TGGACTGGTAAATGAATAACATGCA	diphosphorylase
BRADI4G43900.1	ACATCAATTGCCAAT*TGGACTTATCATAGATCTTCGGTGT	antiporter

EC1

BRADI4G13280.1	GGTACTGAACTTGTACGTGTT*CAGATAAGGGAACACAAGGC	unknown protein
BRADI1G21270.1	CATTGTGCCCATTGAGAAGCT*CAGATGTTGTCCAAATATAA	helicase
BRADI1G46975.1	CATGAAGTACATGCTAATTTT*CAGAAAAACAACAAGTTCAA	RRM
BRADI2G24790.1	TTCCCGTCTTATTTACTGTAT*CAGAATCGTTATATTATGAT	NAM-like protein
BRADI4G13267.1	GAGCTGAAGACTTTGTGCATG*CAGAAATCACCATGACAGTA	unknown protein
BRADI3G34260.1	AGGAAGTGAGGAACCCAGCAG*CAGATGATGCATGTGTCCAC	acyltransferase
BRADI2G15630.2	TTCAATCGACAGCTCTTCCAG*CAGATATAGCTTCTTAAGCA	unknown protein
BRADI1G47920.1	CAGGCCTTCATGCTCCAGCAG*CAGATGATGATGGCCAGCCA	unknown protein
BRADI4G11580.1	CTTCACTTCTATTCGGCATTG*CAGATGACATGGACTTTGCC	aminotransferase-like

EC2

BRADI5G08960.1	CTTCCTGACGTTCCAC*CAGGAATGGCTGATGCAGTGAAC	unknown protein
BRADI3G11036.1	TGATGGTTTTTAGAAC*CAGGACGTTGGGTAGTGTGAAGC	unknown protein
BRADI2G28900.1	TGATGATGATGGGTTC*CAGGATCTCGACTTGATGGGCCT	unknown protein
BRADI2G28910.1	ATCCACCACCCAAGGC*CAGGATCGATCGAGCAGTGCCGT	MYB
BRADI4G42320.1	TGTGCATGTAAAATAC*CAGGATACGTACGTGTTGGATGC	dehydrogenase
BRADI2G08310.1	ACGACGCCTCCAGCAG*CAGGAGGATCAGGACGACGACGG	galactosyltransferase
BRADI3G38020.1	GCCGCCGGTCCAGCAG*CAGGACTCTTCCGGTGCTGTGGA	steroid dehydrogenase
BRADI5G26820.1	TGCTTGCACACAGATG*CAGGACACTGGAATTTTGCAAGA	antiporter
BRADI2G13610.1	GAACCAAGACCAGCAG*CAGGTAGGGGACGAACTATGGGC	unknown protein
BRADI1G62940.1	AATTAATGAGGTAAAC*CAGGTTGCTCAGCTCCGGATCAT	ribosomal protein

EC3

BRADI2G00320.1	TATCTGTCTTTCGGATCT*TCAGATGAACAGTTGCTAGA	putative importin-α
BRADI2G47100.3	GTGTTTATTGTTGTAGGT*TCAGATCATAGTCCAATCTG	myosin phosphatase
BRADI4G29680.1	TATATGTGTATATATTAT*TCAGATCTGGGGACCTTAGT	annexin-like protein
BRADI3G03112.1	GACCGAGAAACTTTATAT*TCAGATGGAACTGTGCGACC	WEE1-like kinase
BRADI1G43220.1	GCCCAAATAAGAAAACCT*TCAGACTGACTCAACGCTGC	constans-like protein
BRADI3G02870.1	GTCATGAACGTATTATTT*TCAGACTAATGTTGTGAGAT	mRNA splicing factor
BRADI1G31450.1	TGATATCTGTATCACCGT*TCAGACGGTCTTGACCGTAC	glutaredoxin like
BRADI3G29750.1	GTTTTTTGCTGCATTCTT*TCAGACCATACTGGCATTGT	serine kinase
BRADI3G51650.1	CATCGGAACCGACAATTG*TCAGACAACTTGGTTTCGAT	zinc finger protein
BRADI5G18180.1	CTCATTGGGATTGGAGGG*TCAGACCATCTACCAGAGAA	STE kinases

BRADI1G38010.1 TCAGTTTTTGGAACTTTG*TCAGACGATGAGATATACTT ribosomal protein
BRADI2G08890.1 TTCCTAGTTTCTTGACTG*TCAGACTGCTTTCGGCTTTT protein kinases

EC4
BRADI1G66627.1 TTATTTATTATTCG*TTCGACTGTGCATCATTTGGAC palmitoyltransferase
BRADI3G38010.1 TACCAAATGTTGTG*TTCGATCCTGCACTTAGAGAAA phosphoglycerate mutase
BRADI2G35820.1 CAACTTATAAGTCT*TTCGATATAAACCTTCCTGCTG cullin-like protein
BRADI1G55020.1 TACTATGTTTATCT*TTCGACTCATCTTCTGTCATCA RNA-binding protein
BRADI4G13940.1 TTTTTTTTGTAAAT*TTCGACGCATGTGCTAATGTCG serine-tRNA ligase
BRADI5G10800.1 GTGTAGTTAGTGCT*TTCGACATTACATGGTTTGTCG translocon
BRADI2G15470.1 GGAAGTGCGCGGCT*TTCGACCTGGCCCCGAAGCTGA unknown protein

Figure S8. **Endonucleolytic cleavages identified in the DW and DC library.** Transcripts with endonucleolytic cleavages shared different conserved motifs. Conserved motifs, at the endonucleolytic cleavage sites, were marked with gray background. Cleavage sites were indicated by the asterisk. The putative functions of all the transcripts with endonucleolytic cleavages were shown. The majority of the detected endonucleolytic cleavages were also found in corresponding biological replicate PARE libraries. Underlined gene names denoted endonucleolytic cleavages that were not detected in corresponding biological replicate PARE libraries.
EW: endonucleolytic cleavage groups only found in the DW libray;
EC: endonucleolytic cleavage groups only found in the DC library;
GONG: endonucleolytic cleavage groups found in both DW and DC library.
